# Application of Narrative and AI-Assisted Follow-Up After Voluntary Medical Male Circumcision: Multicenter, Double-Blind, Prospective, Randomized Controlled Trial

**DOI:** 10.2196/68573

**Published:** 2025-11-17

**Authors:** Linfeng Wang, Yueqiang Peng, Gaojie Zhang, Yong Huang, Guo Yang, Yu Jiang, Qiao Xu, Jiang Yu, Jiajia Jin, Hong Qiao, Qingyu Wu, Ziling Wei, Wei Tang, Jiayu Liu

**Affiliations:** 1 Department of Urology, First Affiliated Hospital of Chongqing Medical University, No. 1 Youyi Road, Chongqing, Yuzhong District, 400016, China, 86 18696682178; 2 Department of Urology, Peking Union Medical College Hospital, Beijing, China; 3 Department of Urology, First Hospital of Jilin University, Changchun, China; 4 Department of Urology, Yongchuan Hospital of Chongqing Medical University, Chongqing, China; 5 School of Nursing, Xiangya Medical School, Sichuan, China; 6 Department of Psychiatry, Chongqing Cancer Hospital, Chongqing, China; 7 School of Nursing, Chongqing Medical University, Chongqing, China; 8 School of the First Clinical Medicine, Chongqing Medical University, Chongqing, China

**Keywords:** artificial intelligence, AI, voluntary medical male circumcision, VMMC, patient-clinician communication, narratives, anxiety

## Abstract

**Background:**

Postoperative anxiety following voluntary medical male circumcision (VMMC) poses a significant health challenge, with limited telemedicine access and inadequate communication compromising recovery and adherence. Narrative-based interventions have shown promise in reducing psychological distress in other contexts, and large language models may enable automated follow-up, but their role in VMMC care remains underexplored.

**Objective:**

We evaluated the effect of a narrative-enhanced tool (NET) on anxiety, sleep quality, quality of life, and pain management and identified risk factors for postoperative anxiety. We also assessed the feasibility of an artificial intelligence–assisted consultation (AAC) system in improving follow-up efficiency.

**Methods:**

From October 1, 2023, to April 29, 2024, patients aged ≥15 years undergoing VMMC were recruited and randomized 1:1 to a standardized risk tool (SRT) or NET group. In addition to the routine postoperative communication, the NET group received a narrative video highlighting positive recovery experiences. Both groups accessed an AAC chatbot for automated follow-ups. Primary outcomes were anxiety levels measured by the 7-item Generalized Anxiety Disorder scale (GAD-7), sleep quality measured by Pittsburgh Sleep Quality Index, quality of life measured by 3-level EuroQoL 5D questionnaire, and pain levels measured by Numerical Rating Scale. Secondary outcomes included analgesic use, satisfaction, and health care worker efficiency. Repeated measures ANOVA assessed trends and regression identified risk factors for anxiety.

**Results:**

Between October 1, 2023, and April 29, 2024, 671 eligible participants were enrolled, with 388 completing the 30-day follow-up (SRT group: n=189, mean age 26.21, SD 3.69 years; NET group: n=199, mean age 26.41, SD 3.56 years; *P*=.60). Both groups exhibited increased anxiety levels, diminished quality of life, and poorer sleep quality during the 30-day postoperative period. However, compared to SRT, the NET group demonstrated lower GAD-7 scores (7.06, SD 2.73 vs. 9.95, SD 3.50; *P*<.001), improved sleep quality (12.29, SD 3.57 vs 13.20, SD 3.54; *P*=.01), higher quality of life scores (0.87, SD 0.07 vs 0.84, SD 0.09; *P*<.001), more regular analgesic use (154/173, 89.02% vs 100/169, 59.17%; *P*<.001), reduced opioid consumption (5/173, 2.89% vs 25/169, 14.79%; *P*<.001), and higher pain medication satisfaction (4.21, SD 0.69 vs 3.76, SD 0.97; *P*<.001). Multivariate analysis identified SRT assignment, inability to recall opioid risk levels, hematoma, swelling, and pain as independent risk factors for elevated GAD-7 scores. Implementation of the AAC substantially reduced health care worker follow-up time (2.34, SD 1.95 min vs 7.85, SD 2.65 min; *P*<.001).

**Conclusions:**

The study demonstrates that narrative is effective in reducing anxiety, improving quality of life, and improving pain management post-VMMC. The integration of artificial intelligence into clinical follow-up protocols has the potential to enhance health care worker efficiency without compromising patient satisfaction.

## Introduction

Since OpenAI released ChatGPT, generative artificial intelligence (AI) has become a focal point of global attention [1,2]. In China, the adoption of virtual medical care, such as “intelligent health care,” for delivering information is becoming widespread [3,4]. Kong et al [5] evaluated 3 large language models (LLMs) as counseling tools for *Helicobacter pylori* infection in multiple languages. He et al [6] indicated that LLMs can offer valuable medical guidance to autistic patients and may surpass physicians in empathy. Additionally, Hua et al [7] created an LLM focused on traditional Chinese medicine, demonstrating AI’s potential to enhance clinical decision-making in diagnostics and treatments. Moreover, evidence suggests that AI can be trained into advanced language models (also known as an intelligent agent), functioning as personal virtual assistants for anyone with internet access, which can process free text prompts, understand semantics, respond appropriately, and maintain context in personalized conversations [8]. They can deliver accurate answers for symptom assessment and classification [9-11], facilitating timely communication of medical information [12] and promoting “intelligent health care.”

Anxiety, a psychological state characterized by apprehensive expectations or fear, is among the most commonly experienced psychiatric symptoms [13]. Data from the Young and Well Cooperative Research Center suggest that its prevalence is increasing among individuals aged 16 to 25 years, posing a challenge to global public health [14]. In clinical work, we found that inadequate clinician–patient communication leads to a lack of awareness about pain management among patients who underwent voluntary medical male circumcision (VMMC) [15], eventually resulting in emotional disorders or substance abuse [16,17]. This prompted us to explore more effective methods of postoperative communication and follow-up. Rocha et al [18] emphasized the importance of narratives in the context of terminating a wanted pregnancy, which had positive effects on women’s mental health. Katz et al [19] provided support for the effects of narrative risk communication in influencing feelings of cancer risk through ease of imagination. Collectively, narratives with detailed personal experiences allow listeners to concentrate more on the content [17], which may enhance postoperative patients’ awareness of surgical risks.

To our knowledge, researchers have focused on addressing anxiety during the perioperative period of circumcision through distraction, virtual reality, or music therapy [20-22]. However, few studies have assessed the effects of narrative communication in alleviating anxiety in patients after circumcision. Therefore, we conducted this multicenter, prospective, double-blind, randomized controlled trial to evaluate the effectiveness of narrative-enhanced communication by assessing the anxiety scores of the participants. Additionally, we introduced an agent-based AI-assisted follow-up tool to assess its value for the first time in a trial.

## Methods

### Study Design

This multicenter, double-blind, randomized clinical trial was conducted in 5 urology departments in Chongqing, China. The trial was conducted from October 1, 2023, to April 29, 2024, and has been registered in the Clinical Trial Registry (No. ChiCTR2300076099). This study followed the Consolidated Standards of Reporting Trials eHealth (CONSORT-EHEALTH) reporting guidelines [23].

### Ethical Considerations

The primary objective of this trial was to improve postoperative communication following VMMC. The trial was approved by the Ethics Committee of the First Affiliated Hospital of Chongqing Medical University (2023‐264) and followed the principles of the Declaration of Helsinki. Written informed consent was obtained from all male participants aged ≥15 years or their guardians. Prior to the analysis, all patient identifiers (including medical record numbers, names, and addresses) were removed to protect participants’ privacy and personal information. In addition, all participants received transportation subsidies.

### Participants

The participants in this research were recruited continuously, and the inclusion criteria included male patients (≥15 y) who received VMMC for phimosis or redundant prepuce and who could use smartphones to complete web-based questionnaires. The exclusion criteria included a history of mental disorders, baseline 7-item Generalized Anxiety Disorder (GAD-7) score ≥10, unable to take opioids or non-steroidal anti-inflammatory drugs for any reason, and unable to provide written informed consent.

### Randomization

Eligible participants were randomly assigned (1:1) to the standardized risk tool (SRT) group and the narrative enhanced tool (NET) group and received the SRT or NET manual (Multimedia Appendix 1). Randomization was achieved using a computer-generated process. We used Python’s random library to generate random numbers for each participant. Specifically, the random.randint(1, 100) function assigned a random integer between 1 and 100 to each patient. Patients with numbers 1‐50 were assigned to the treatment group, while those with numbers 51‐100 were assigned to the control group. The random information was stored in a password-protected Excel file, accessible only to the principal investigator, who was not involved in the consent or assessment process. The patients and clinicians were blinded to the randomization process.

### Procedure

A meeting was held before the start of the trial, which confirmed the specific procedures of the entire trial, ensured that each experimental center had rooms for participants and investigators to meet privately, and utilized the same type of circumcision device, thereby ensuring consistency in the multicenter clinical practice. We assigned doctors with extensive experience in performing circumcisions at each center. Moreover, we minimized unnecessary hospital-based bias in the trial by providing subsequent training and establishing independent trial center researchers. Although the trial started using AI-assisted follow-up on October 10, 2023, the staff involved remained unchanged.

The specific process of the trial included a 2-day screening period (varying by hospital), 1 day for surgery, and a 30-day follow-up period (Figure S1 in Multimedia Appendix 2). Before surgery, investigators will take participants into a private meeting room for preoperative communication, which, as required, includes the following: the surgical procedure of circumcision, the purpose and necessity of the surgery, and the potential risks associated with the surgery. At the same time, the investigators will instruct the participants on how to correctly fill out the online questionnaire to ensure validity and reliability. All participants were required to return to the room after the circumcision for 30 min vital signs monitoring. Meanwhile, investigators would provide different manuals based on the assigned groups. Both manuals provided general guidelines on postoperative recovery and strategies for coping with postoperative complications. The contents of both manuals are summarized as follows:

- SRT: In addition to the standard postoperative and discharge instructions for outpatient surgery, all participants received fact-based risk information, which contained information on the benefits, side effects, and risks of analgesics (including opioid medications independently prescribed by a psychiatrist).
- NET: A narrative-enhanced audiovisual communication tool was employed. The tool was designed through a combined process, in which our research team demonstrated postoperative risks and information on the use of analgesics and then combined this with video narratives that included real stories from patients with opioid use disorder (OUD). The NET incorporated 4 distinct narratives. These narratives included the reasons for initial exposure to opioids (including chronic pain). Meanwhile, a professional film crew documented these narratives, which had previously appeared in news media—enhancing both the readability and script reliability of the NET intervention. All these narratives were from speakers in China. The combined video had a length of approximately 10 minutes [24,25]. The intervention occurred within 30 minutes post-surgery, with the investigators confirming that the manuals had been thoroughly administered.

Follow-ups were conducted at the corresponding time points after circumcision (Figure S1 in Multimedia Appendix 2). In the early stages of the trial, we regularly sent online survey questionnaires to participants via the 2-way texting consulting (TTC) and conducted online dialogue with participants. From the midpoint of the trial, follow-up was conducted with the assistance of the AI. We constructed a TTC platform based on SMS to provide patients with more efficient telemedicine services. Researchers have indicated that AI algorithms can potentially be applied to perform tedious tasks that are time-consuming and devoid of intellectual challenges [26]. In this study, we also recognized this and introduced “intelligent agents” to optimize the follow-up procedure from October 10, 2023.

AI-assisted consulting (AAC) is implemented through an application programming interface that integrates a generative intelligent agent with WeChat, which is the most widely used mobile application in China. Through AAC, staff can proactively initiate conversations with patients, and patients can interact with the agent to receive information and support (Figure S2 in Multimedia Appendix 2). The model leverages a wide range of high-quality medical datasets to enhance its specialized clinical knowledge base. Moreover, it complies with China’s first technical standard for medical AI systems and has been validated through the MedBench evaluation framework [27], ensuring expert-level clinical performance, protocol standardization, and certified safety in medical applications. AAC integrates user health records by constructing patient profiles through backend data, enabling personalized dialogue. The accuracy of its domain-specific knowledge—covering areas such as VMMC and mental health disorders—has been prevalidated. Once deployed as an AI agent, the AAC platform records patient consultations and extracts key information such as comorbidities, thereby facilitating reinforcement learning tailored to individual patients. All AAC responses are reviewed weekly by our follow-up team, with a verified response accuracy exceeding 90%.

For this trial, the AAC platform’s medical knowledge is current as of October 2023. Its refusal policy includes the following: (1) declining to respond to inquiries involving personal privacy or safety concerns, (2) maintaining neutrality on questions requiring subjective judgment, (3) refraining from answering queries beyond its knowledge base and training scope, (4) avoiding topics that may provoke controversy or conflict, and (5) not intentionally refusing to respond to user questions. The AAC platform employs Advanced Encryption Standard high-strength encryption and HTTP (hypertext transfer protocol) Secure protocols to ensure secure data storage and transmission. Internal personnel must undergo strict identity verification and receive explicit access authorization before handling user data. All operations are traceable through audit logs. Access to sensitive data requires multiparty authorization to mitigate the risk of single-point data leakage.

### Outcomes

The primary outcome was the difference in change in anxiety level, sleep quality, quality of life, and pain level using the GAD-7, Pittsburgh Sleep Quality Index (PSQI), 3-level EuroQoL 5D questionnaire (EQ-5D-3L), and multiple tools–assisted Numerical Rating Scale (ma-NRS). The GAD-7 scale consists of 7 items that assess the anxiety levels of participants over the past 2 weeks, with higher scores indicating greater anxiety (0‐21 points) [28,29]. GAD-7 had been validated in various cultures, including China, demonstrating good reliability and validity. PSQI and EQ-5D-3L have been used to assess sleep and quality of life, respectively [30,31]. The PSQI consists of 25 items measuring 7 subscales that assess sleep quality, and higher scores indicate worse global sleep quality (0‐21 points). The EQ-5D series is a widely used generic utility measure globally, with EQ-5D-3L being easily understood and requiring little response burden. A version of the EQ-5D-3L based on urban residents in China has been established [32], with health utility scores calculated using the time trade-off value (0‐1 points), where lower scores indicated poorer quality of life [33]. Ma-NRS aims to enhance the accuracy of existing NRS through visual prompts and textual descriptors. The ma-NRS (0‐10 points) combined NRS-101 [34,35], the Wong-Baker FACES Scale [36], and the 11-point verbal rating scale [37].

The secondary outcomes were the use of analgesics and 2 follow-up methods. Satisfaction scores were measured using a 5-point Likert scale, with higher scores (1‐5 points) indicating greater satisfaction. The time spent by the staff to reply to messages was documented using the backend statistical system. We assessed the risk of OUD for all participants with the opioid risk tool (ORT). Recall of the ORT involved asking during the last online follow-up, “What was your level of OUD risk: (low, medium, high),” and comparing this with the baseline consistency (low: 0‐3, medium: 4‐7, and high: ≥8) [38]. The safety outcomes were adverse events (AEs), including edema, bleeding, and infection, until 30 days after surgery.

### Sample Size

Assuming the effect size of the primary outcome was 0.128, it was determined that 162 participants were needed per group to achieve 80% statistical power at a 5% significance level (2-sided hypothesis test) to detect a minimally meaningful effect. The sample size was increased to 360 to allow for 10% withdrawal and loss to follow-up.

### Statistical Analyses

Statistical analyses were conducted using SPSS 26.0. All the analyses were based on the intention-to-treat principle. In the analyses of primary outcomes, repeated measures ANOVA was used to evaluate the primary outcomes between groups, with time and group as independent variables. Greenhouse-Geisser and Bonferroni corrections were applied where appropriate. Simple effects analysis was used to determine whether the differences in the primary outcomes between the 2 groups at each time point were significant. Further discussion of the independent risk factors leading to anxiety was conducted using univariate and multivariate linear regression analyses. For the sensitivity analysis regarding education level and AAC usage, we performed subgroup analysis by education level and AAC, followed by mediation analysis to evaluate the mediating and indirect effects of education level and AAC use. The *χ*^2^ test was used to compare the occurrence of AEs. For secondary outcomes, *t* or *U* tests and chi-squared test were the primary statistical methods employed.

## Results

### Participants

**Figure 1.**
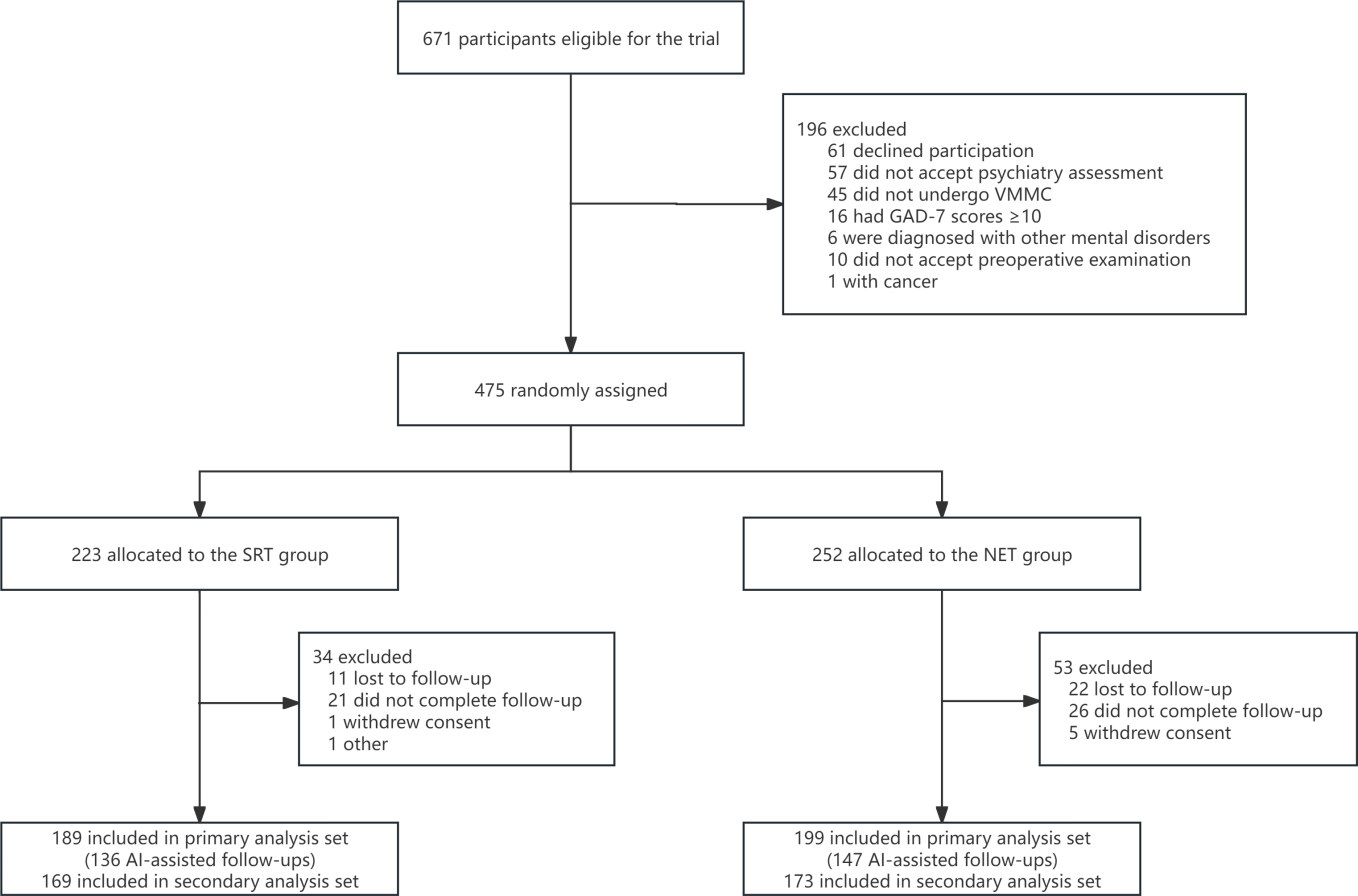
CONSORT flow diagram of participant allocation to NET and SRT groups with exclusion counts at each study stage. AI: artificial intelligence; CONSORT: Consolidated Standards of Reporting Trials; NET: narrative enhanced tool; SRT: standardized risk tool; VMMC: voluntary medical male circumcision.

From October 1, 2023, to April 29, 2024, a total of 671 participants qualified for inclusion during their first outpatient visit, with 196 excluded for various reasons (Figure 1). Of the 475 participants randomly assigned to the SRT and NET groups, 189 and 199 participants completed the 30-day follow-up and were included, respectively. Approximately 88.17% of the participants used analgesics, and 72.94% of the participants were followed up using AAC (Figure 1). Table 1 presents the comparison of baseline characteristics between the 2 groups.

**Table 1.** Comparison of baseline characteristics between SRT^a^ and NET^b^ groups.^c^

	SRT group (n=189)	NET group (n=199)	*P* value
Age (years), mean (SD)	26.21 (3.69)	26.41 (3.56)	.60
BMI, kg/m^2^, mean (SD)	22.63 (3.57)	22.80 (3.58)	.63
Smoking status, n (%)	75 (39.68)	61 (30.65)	.06
Alcohol status, n (%)	53 (28.04)	60 (30.15)	.65
Education, n (%)			.05
Primary school graduate or less	2 (1.06)	0 (0)	
Junior school graduate	7 (3.7)	15 (7.54)	
High school graduate	53 (28.04)	73 (36.68)	
College graduate	53 (28.04)	94 (47.24)	
Postgraduate or above	24 (12.7)	17 (8.54)	
Baseline GAD-7^d^, mean (SD)	2.12 (0.92)	2.22 (1.14)	.35
Baseline PSQI^e^, mean (SD)	4.95 (2.06)	5.04 (2.13)	.68
Baseline QoL^f^, mean (SD)	0.95 (0.02)	0.95 (0.04)	.67

a SRT: standardized risk tool.

b NET: narrative enhanced tool.

c Data are presented as n (%) of participants unless otherwise noted. Continuous data, expressed as mean (SD), were compared using the *t* test or Mann-Whitney *U* test. Outcomes expressed as percentages of patients with each outcome were compared between the 2 groups using the *χ*2 test or the Fisher exact test.

d GAD: Generalized Anxiety Disorder.

e PSQI: Pittsburgh Sleep Quality Index.

f QoL: quality of life.

### Primary Outcome: Changes in Anxiety, Sleep Quality, Quality of Life, and Pain

**Figure 2.**
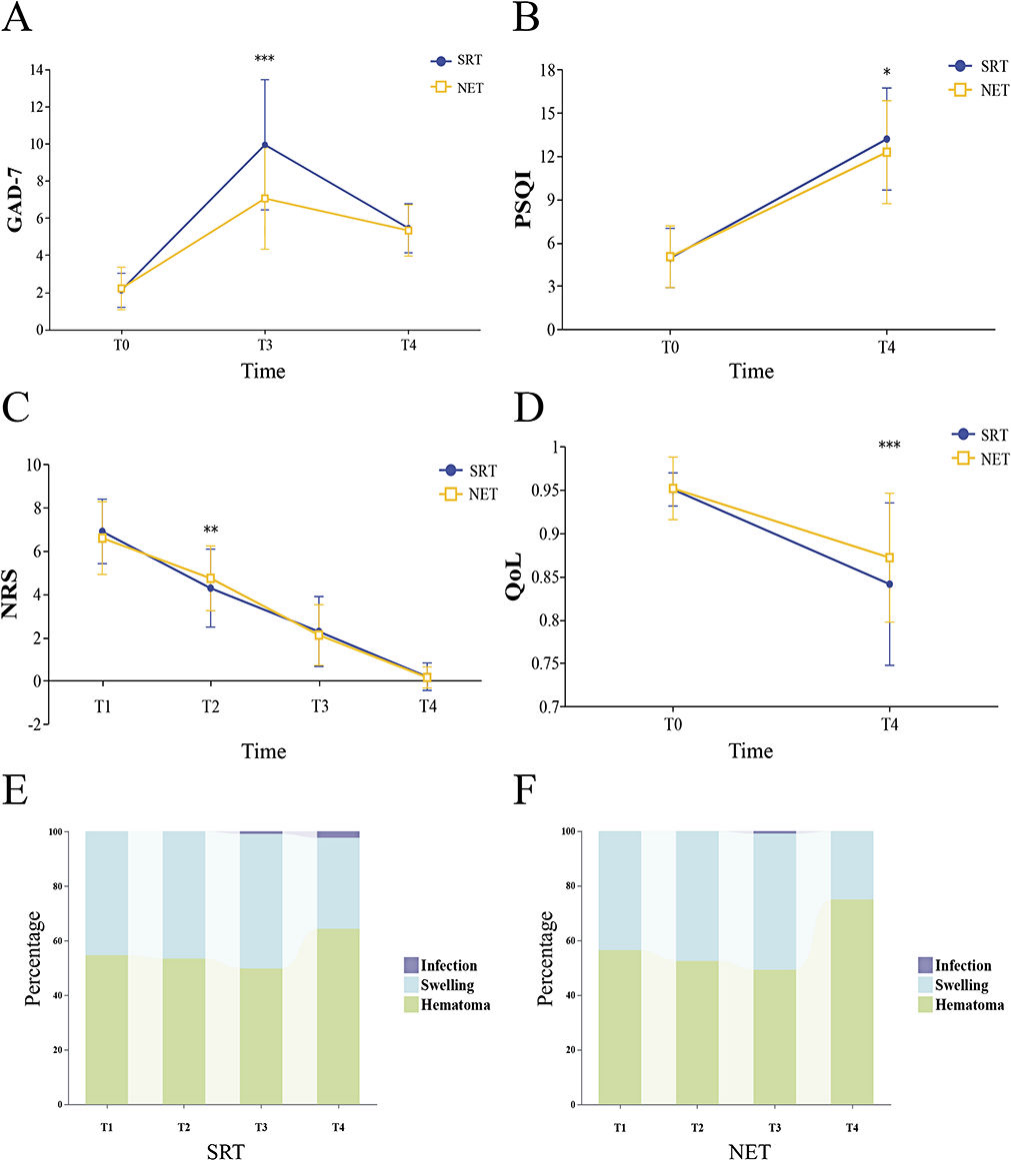
Comparison of GAD-7, PSQI, NRS, and QoL scores and adverse event rates between SRT and NET groups at different time points after VMMC. (A–D) The scores of the GAD-7, PSQI, ma-NRS, and QoL scales measured at each time point for the 2 groups, respectively. (E-F) The proportions of infection, swelling, and hematoma reported at each time point compared to the total complications. The statistical significance results were derived from a simple ANOVA. GAD-7: 7-item Generalized Anxiety Disorder scale; NET: narrative enhanced tool; NRS: Numerical Rating Scale; PSQI: Pittsburgh Sleep Quality Index; QoL: quality of life; SRT: standardized risk tool; VMMC: voluntary medical male circumcision. *P<.05, **P<.01, ***P<.001.

Figure 2A–D illustrates primary outcomes measure change over time; specific data are shown in Tables S1 and S2 in Multimedia Appendix 2. These results indicated that time had a significant main effect on the 4 primary outcomes. GAD-7 scores presented a temporary uptrend first and then a decline (2.12, SD 0.92 vs 9.95, SD 3.50 vs 5.46, SD 1.32; 2.22, SD 1.14 vs 7.06, SD 2.73 vs 5.34, SD 1.38; *F*_2,772_=920.78; *P*_time_<.001), and the NET group had significantly lower GAD-7 scores than those in the SRT group at T3 (7.06, SD 2.73 vs. 9.95, SD 3.50, *P*<.001). PSQI scores increased at day 30 (T4) (4.95, SD 2.06 vs 13.20, SD 3.54; 5.04, SD 2.13vs 12.29, SD 3.57; *F*_1,386_=1352.07; *P*_time_<.001), indicating deteriorated sleep quality. However, the SRT group demonstrated comparatively worse sleep quality at T4 (13.20, SD 3.54 vs 12.29, SD 3.57; *P*=.01). During the 30-day postoperative follow-up, ma-NRS scores decreased over time (6.91, SD 1.49 vs 4.29, SD 1.80 vs 2.29, SD 1.62 vs 0.20, SD 0.35; 6.60, SD 1.68 vs 4.74, SD 1.50 vs 2.12, SD 1.40 vs 0.16, SD 0.49; *F*_3,1158_=1693.83; *P*_time_<.001). However, during the early postoperative phase (T2), the NET group showed significantly higher pain levels (4.74, SD 1.50 vs. 4.29, SD 1.80; *P*=.008). QoL scores decreased at the end point (T4) (0.95, SD 0.02 vs 0.86, SD 0.09; 0.95, SD 0.04 vs 0.86, SD 0.08; *F*_1,386_=441.73; *P*_time_<.001), and the SRT group demonstrated a more pronounced reduction at T4 (0.84, SD 0.09 vs. 0.87, SD 0.07; *P*<.001). GAD-7 and ma-NRS scores involved ≥3 measurement points, and Greenhouse-Geisser–corrected sphericity tests were conducted.

### Secondary Outcomes

#### Adverse Events

Figure 2E and F demonstrated results of safety outcomes, showing no difference in the incidence of perineal hematoma, penile swelling, and incision infections between the 2 groups at each time point; specific data are shown in Table S2 in Multimedia Appendix 2. Almost all participants experienced a perineal hematoma (T2). Up to 87.30% (165/189) and 90.45% (180/199) of the participants in the SRT and NET groups, respectively, experienced perineal swelling. By the 30th day of the trial (T4), hematoma and swelling decreased to approximately 5%‐15% (Table S2 in Multimedia Appendix 2). Incision infection rate among the participants included in the analysis was 1.29% (5/388), and incision infections were first observed on the 14th postoperative day. After reviewing the chat data, we found that the earliest reported incision infection occurred on the 10th postoperative day in the SRT group, with the remaining 4 cases occurring on the 13th and 22nd postoperative days (SRT group) and the 11th and 13th postoperative days (NET group). Notably, one unexpected complication occurred in this study, in which a patient experienced incision dehiscence at home. Fortunately, he quickly contacted clinicians through the follow-up platform and received timely treatment.

#### Risk Factors of Anxiety

GAD-7 levels peaked on the 14th day of follow-up (T3), which also represented the most pronounced negative emotions observed among participants in this trial. Results of the linear regression analysis indicated significant relationships between grouping, ORT recall, regularity of analgesic use, opioid usage, swelling, hematoma, NRS scores, and satisfaction with analgesic effects with GAD-7 scores (T3) (Table S3 in Multimedia Appendix 2). The result of the multivariable linear regression using these 7 factors is presented in Table 2. Grouping (β=−.382, 95% CI −3.150 to −2.048; *P*<.001), ORT recall (β=−.103, 95% CI −2.150 to −0.333; *P*<.001), swelling reported on the 14th postoperative day (β=.456, 95% CI 2.604 to 3.654; *P*<.001), hematoma (β=.254, 95% CI 1.215 to 2.265; *P*<.001), and NRS scores (β=.097, 95% CI 0.048 to 0.379; *P*=.01) were identified as independent risk factors for GAD-7 scores (T3) (Table 2).

**Table 2.** Multivariate regression analysis of GAD-7^a^ (T3) scores using risk factors identified from univariate regression results^b^^,^^c^.

	B	SE	β	*P*	95% CI	VIF^d^
Group^e^	−2.599	0.281	−.382	<.001^f^	−3.150 to −2.048	1.179
Regularity of analgesic	0.048	0.321	.006	.882	−0.582 to 0.677	1.179
ORT^g^ recall	−1.242	0.463	−.103	<.001^f^	−2.150 to −0.333	1.027
Opioid used	0.249	0.475	.021	.60	−0.683 to 1.181	1.080
Swelling (T3)	3.129	0.268	.456	<.001^f^	2.604 to 3.654	1.052
Hematoma (T3)	1.740	0.268	.254	<.001^f^	1.215 to 2.265	1.056
Pain (T3)	0.214	0.084	.097	.01^f^	0.048 to 0.379	1.014

a GAD-7: 7-item Generalized Anxiety Disorder scale.

b Regressions were adjusted for all risk factors except itself.

c *F*_7,334_=50.838; *P*<.001.

d VIF: variance inflation factor.

e The dummy variable setting for “group”: SRT=0; NET =1.

f *P<*.05

g ORT: opioid risk tool.

#### Pain Management

Table 3 presents results for other secondary outcomes. Use of analgesic medication was reported by 89.42% (169/189) of patients in the SRT group and 86.93% (173/199) of patients in the NET group, with no significant difference between the 2 groups (*P*=.45). Of note, the NET group had a significantly higher proportion of participants with regular analgesic use (154/173, 89.02% vs 100/169, 59.17%; *P*<.001), as well as greater satisfaction with pain medication (4.21, SD 0.69 vs 3.76, SD 0.97; *P*<.001). Furthermore, the SRT group demonstrated a significantly higher rate of opioid use, with 14.79% (25/169) of participants using opioids compared to only 2.89% (5/173) in the NET group (*P*<.001). At the end point, there was no difference in ORT recall rates between the SRT and NET groups (166/189, 87.83% vs 186/199, 93.47%; *P*=.06).

**Table 3.** Differences in postoperative analgesic drug usage between SRT^a^ and NET^b^ groups and comparison across follow-up groups (AAC^c^ vs TTC^d^) after VMMC.^e^

	SRT group (n=189)	NET group (n=199)	*P* value
Analgesic medication used, n (%)	169 (89.42)	173 (86.93)	.45
ORT^f^ recall, n (%)	166 (87.83)	186 (93.47)	.06
Satisfaction score of analgesic medication, mean (SD)	3.76 (0.97)	4.21 (0.69)	<.001
Regularity of analgesic medication, n/N (%)	100/169 (59.17)	154/173 (89.02)	<.001
Opioid used, n/N (%)	25/169 (14.79)	5/173 (2.89)	<.001
Follow-up method, n (%)			.67
AAC^g^	136 (71.96)	147 (73.87)	
TTC^h^	53 (28.04)	52 (26.13)	

a SRT: standardized risk tool.

b NET: narrative enhanced tool.

c AAC: artificial intelligence–assisted consulting.

d TTC: 2-way texting consulting

e Data are presented as n (%) of participants unless otherwise noted. Continuous data, expressed as mean (SD), were compared using the *t *test or Mann-Whitney *U* test. Outcomes expressed as percentages of patients with each outcome were compared between the 2 groups using the *χ*2 test or the Fisher exact test.

f ORT: opioid risk tool.

g Time spent per interval (min): 2.34 (SD 1.95; *P*<.001); Follow-up satisfaction score: 4.10 (SD 1.03; *P*=.16).

h Time spent per interval (min): 7.85 (SD 2.65; *P*<.001); Follow-up satisfaction score: 4.31 (SD 0.80; *P*=.16).

#### AAC Use

Most participants utilized AAC, with 71.96% (n=136) of SRT group and 73.87% (n=147) of NET group (Table 3). Participants reported similar satisfaction scores for 2 follow-up methods (4.10, SD 1.03 for AAC and 4.31, SD 0.80 for TTC; *P*=.16). Compared to the TTC group, the time spent per patient per session by follow-up staff in the AAC group was significantly reduced, averaging 2.34 (SD 1.95) minutes, while the TTC group averaged 7.85 (SD 2.65) minutes (*P*<.001).

### Sensitivity Analysis

#### Education Level Does Not Affect the Effectiveness of NET

Given that education level may influence patients’ comprehension of risk information and thus potentially affect the reliability of NET-related outcomes in pain management, we conducted sensitivity analyses. Table S4 in Multimedia Appendix 2 presents subgroup analyses stratified by education level, demonstrating that the intervention effects of NET on the regularity of analgesic use, analgesic utilization rate, opioid use rate, ORT recall, and satisfaction with analgesic use were not influenced by education level (all *P* for interaction ≥.05). Both mediation and path analyses further confirmed that education level was neither a mediator nor an indirect factor in the association between NET and pain management–related outcomes (Table S5 in Multimedia Appendix 2, all *P* indirect >.05; Table S6 in Multimedia Appendix 2, all *P* for Exposure→Mediator >.05).

#### AAC Does Not Affect the Effects of NET on GAD-7, PSQI, QoL, and NRS Scores

Since October 2023, we had used AAC to follow up newly enrolled patients. To eliminate potential confounding effects of different follow-up methods on the primary outcome conclusions, we performed subgroup analyses stratified by follow-up modality (TTC/AAC). The results demonstrated that the primary outcomes of this study—namely, the effects of NET on GAD-7, PSQI, QoL, and NRS scores in post-VMMC patients—were not influenced by the follow-up method used (Table S7 in Multimedia Appendix 2, all *P* for interaction ≥.05). Similarly, mediation and path analyses indicated that AAC was neither a mediator nor an indirect influencing factor in the relationship between NET and the primary outcomes (Table S8 in Multimedia Appendix 2, all *P* indirect >.05; Table S9 in Multimedia Appendix 2, all *P* for Exposure→Mediator >.05).

## Discussion

### Principal Findings

In this randomized controlled double-blind multicenter trial, we found that patients experienced anxiety after VMMC due to pain and AEs. We observed that after VMMC surgery, patients’ anxiety levels first increased and then decreased within 30 days postoperatively, and they all experienced poor sleep quality and decreased quality of life. However, in contrast to participants who received SRT postoperative communication, those who received NET communication had relatively lower GAD-7 scores, lower PSQI scores, and relatively better quality of life within 30 days post surgery. Furthermore, participants in the NET group performed better in the analysis set for analgesic medication use. Moreover, as an exploratory method, AI-assisted follow-up significantly reduced the time health care workers spent on follow-up tasks. Finally, the results of sensitivity analysis indicated that the effects of NET post-VMMC were not influenced by education level or different follow-up methods.

### Comparison to Prior Work

Narratives improve the dissemination of health information by capturing people’s attention and “conveying” their psychological states [39]. Results of the present study indicated that AEs following VMMC led to the development of anxious mood in patients during the recovery period and that anxious mood also improved after the symptoms subsided. Clearly, NET did not lead to differences in the incidence of complications between the 2 groups; therefore, we speculated that incorporating NET might have provided VMMC participants with a clearer understanding of the risks or self-limiting complications they were about to face and enabled them to be better psychologically prepared, ultimately reflecting improved scale scores and analgesic medication use. These findings are consistent with earlier research results [17,40].

Owing to advances in technology and surgical instruments, postoperative pain, swelling, and hematoma following VMMC are increasingly well managed, yet we seem to neglect the perspectives of patients regarding these AEs [41-43]. In a follow-up of this study, we found that most participants’ complications had disappeared or improved by 30 days postoperatively. We found it crucial to help patients understand that “these issues were not something I needed to worry about” through NET, as men found it difficult to ignore penile issues, leading to exaggerated concerns and repeated inquiries on the TTC platform. This reaction could be described, albeit imprecisely, as “making a mountain out of a molehill”. Some participants expressed a more long-term concern during the follow-up, specifically regarding fear about sexual function or erectile dysfunction, which was described as “pain during intercourse,” “pulling sensation after erection,” or “discomfort at the frenulum.” We conducted further telephone follow-ups with these participants (the timing varied from February to June) and found that the related symptoms were mostly short-term (16/18).

Chronic pain persisted throughout the recovery period. Unlike surgeries on other parts of the body, pain associated with circumcision in adult patients is particularly unique, as it is closely related to involuntary penile erections during the night and early morning. Comfort and analgesics may have become the primary reliance in the absence of the possibility of administering suppressive functional medications. Previous studies have indicated that narrative communication has a positive effect on risk communication regarding OUD [17]. In this trial, we aimed to encourage participants to use analgesics at regular intervals and adhere to the World Health Organization analgesic ladder through NET. Our results showed that patients in the SRT group had lower ma-NRS scores on the day after the procedure, which may have been related to their eagerness to start using stronger analgesics or increasing dosages after the anesthesia wore off. Nonetheless, there was no significant difference in the overall pain trend between the 2 groups, suggesting that this “over-treatment” of pain issues was not advisable. This has compelled clinicians to reevaluate the issue and prepare individualized plans for patients. Fortunately, AAC used in the clinical setting offers this possibility.

Remarkably, the establishment of the TTC platform was beneficial to clinical work as it provided a rapid means of communication, yet it also increased the burden on health care workers and led to burnout. Consequently, this cohort incorporated an intelligent agent and developed an AI-assisted follow-up platform that guaranteed communication accessibility. It was reported that the medical documents drafted by generative AI had clinical accuracy and text quality similar to those of clinicians, and clinical practitioners only needed to review and make necessary corrections to the AI-generated content [44,45]. According to the results of this study, drafts composed by generative AI improved the work efficiency by approximately 70%. During the process of AAC, we found that drafts were closely aligned with the specific circumstances of patients (Table S10.A in Multimedia Appendix 2) and consistently produced empathetic expressions (Table S10.B in Multimedia Appendix 2). Previous studies also acknowledged that generative AI had a faster response time in interactions with patients, displayed appropriate length and lexical variation, and exhibited greater compassion [9,46,47].

Satisfaction ratings for both follow-up methods averaged at “satisfied (4),” with no statistically significant difference observed. AAC employed a domestic intelligent agent model, which possibly enhanced participants’ preference for it in terms of language expression [12]. On the other hand, the participants were mostly young individuals who may have favored symptom descriptions on the AI-assisted platform due to a sense of shame [48,49]. The explanations generated by the intelligent agent were trained on guidelines and literature reviews, and during the conversations, the model continuously recorded the participants’ characteristics to provide more personalized and safer solutions (Table S10.C in Multimedia Appendix 2). Notably, the AAC group exhibited a larger standard deviation in satisfaction scores, indicating that some patients were dissatisfied with this follow-up method. Such dissatisfaction was not merely a finding of this study. LLMs need improved quality control to enhance the accuracy of information delivered and eliminate misinformation to build trust with patients [47]; LLMs might have also encountered ethical concerns surrounding privacy, fairness, and bias [50]. Furthermore, excessive reliance on AI for tasks such as pathological prediction may have resulted in a decline in physicians’ own skills [26]. These AI-assisted systems may have temporarily provided convenience, but the contact between clinicians and patients would have rapidly decreased, which contradicted the concept of “achieving better clinician–patient communication.” Our real-world experience indicates that we need to maximize the potential benefits of generative AI in providing comfort and completing basic medical inquiries. This not only helped alleviate physician burnout by offloading follow-up tasks, but more importantly, it also enabled the rapid screening of cases that required timely intervention. Moreover, with the assistance of a localized multimodal closed-source generative AI model, the participants’ privacy was ensured. In summary, this constitutes a significant exploration of the application of generative AI within the Chinese health care environment, evidencing its potential to remove communication barriers in clinical follow-up work.

### Limitations

This study has certain limitations. First, bias may have been present in the trial procedures. Since this study was conducted across multiple institutions, although there have been attempts to standardize processes before the trial began, the procedures for anesthesia, surgery, and others ultimately depended on the policies of each institution. Although the use of internationally recognized questionnaires improved the generalizability and validity of the results, the process of filling them out remained subjective. Second, sexual complications may have been underreported in this study; despite receiving little feedback, these factors were highly correlated with anxiety. Furthermore, owing to commercial regulations, the AAC platform was used solely as an auxiliary tool during this specific trial, with the related analyses not being based on RCT rules.

### Future Directions

Before widespread deployment in more challenging clinical environments, generative AI must further improve the accuracy and completeness of text generation and establish comprehensive standards. Lastly, the impact of narrative communication and AI-assisted follow-up on diagnosis, surgical indications, or treatment modalities is limited. However, even a mild reduction in anxiety can significantly enhance the overall patient experience and satisfaction, particularly in the context of postoperative recovery and mental well-being. These findings highlight the potential for integrating such approaches into broader patient care strategies. Future research could focus on expanding the sample size and extending the follow-up period. We plan to conduct further research with a more diverse sample, encompassing different ages, cultural backgrounds, and technological settings. This will help us evaluate the universality of the AI system and its potential applicability in various global contexts.

### Conclusions

The results of this study indicated that narratives could alleviate anxiety in VMMC participants post surgery, while suggesting a potential positive effect on the quality of life. NET has the potential to enhance postoperative communication in outpatient surgical settings. Generative AI specifically designed for postoperative follow-up of VMMC enhanced the efficiency of health care workers. However, if similar solutions are considered for broader clinical applications, further large-scale randomized controlled trials are required for validation.

## Supplementary material

10.2196/68573 Multimedia Appendix 1 Manual files for standardized risk tool (SRT) or narrative enhanced tool (NET) participants.

10.2196/68573 Multimedia Appendix 2 Supplementary tables and figures presenting detailed statistical analyses, subgroup and mediation results, and schematic workflows related to post-VMMC outcomes. VMMC: voluntary medical male circumcision.

10.2196/68573 Checklist 1 CONSORT-eHEALTH checklist (V 1.6.1).

## Abbreviations:

AAC: artificial intelligence–assisted consulting
AE: adverse event
AI: artificial intelligence
CONSORT-EHEALTH: Consolidated Standards of Reporting Trials eHealth
EQ-5D-3L: 3-level EuroQoL 5D questionnaire
GAD-7: 7-item General Anxiety Disorder scale
HTTP: hypertext transfer protocol
LLM: large language model
ma-NRS: multiple tools–assisted Numerical Rating Scale
NET: narrative enhanced tool
NRS: Numerical Rating Scale
ORT: opioid risk tool
OUD: opioid use disorder
PSQI: Pittsburgh Sleep Quality Index
QoL: quality of life
SRT: standardized risk tool
TTC: 2-way texting consulting
VMMC: voluntary medical male circumcision
