# Supplementary material for: Application of Narrative and AI-Assisted Follow-Up After Voluntary Medical Male Circumcision: Multicenter, Double-Blind, Prospective, Randomized Controlled Trial
Source: J Med Internet Res. 2025 Nov 17;27:e68573. doi: 10.2196/68573 (PMC12622909; doi:10.2196/68573)
Supplement: Multimedia Appendix 1 [file jmir-v27-e68573-s001.docx]

**Appendix**

[（一）术后常见症状的处理 1](#_Toc175047614)

[1.术后疼痛 1](#_Toc175047615)

[2.水肿的原因 1](#_Toc175047616)

[3.消肿方法 1](#_Toc175047617)

[（二）血肿怎么会产生的？怎么样处理血肿？ 1](#_Toc175047618)

[（三）伤口液体渗出 1](#_Toc175047619)

[(四)伤口出血 1](#_Toc175047620)

[(五)伤口撕裂 2](#_Toc175047621)

[（六）保持伤口干燥 2](#_Toc175047622)

[(七)预防感染 2](#_Toc175047623)

[常见问题： 2](#_Toc175047624)

[1.包扎多长时间为佳？ 2](#_Toc175047625)

[2.术后能洗澡吗? 2](#_Toc175047626)

[3.小便后尿液浸湿伤口怎么办? 2](#_Toc175047627)

[4.多少天伤口才能愈合? 2](#_Toc175047628)

[5.怎样减少勃起? 2](#_Toc175047629)

[6.缝合钉自行脱落算正常吗? 2](#_Toc175047630)

[7.在15~20天左右大部分的患者应能自行脱完，还有部分患者应个体差异所致缝钉未能完全脱落如何处理? 3](#_Toc175047631)

[8.下包后为什么要穿宽松裤? 3](#_Toc175047632)

[9.术后饮食有什么禁忌? 3](#_Toc175047633)

[10.术后什么时候能同房? 3](#_Toc175047634)

[11.术后能否参加体育活动? 3](#_Toc175047635)

[Postoperative Care after Circumcision 4](#_Toc175047636)

[(A) Management of Common Postoperative Symptoms 4](#_Toc175047637)

[Postoperative Pain 4](#_Toc175047638)

[Causes of Edema 5](#_Toc175047639)

[Methods to Reduce Swelling 5](#_Toc175047640)

[Time for Edema to Subside 5](#_Toc175047641)

[(B) How Do Hematomas Form? How to Handle Hematomas? 6](#_Toc175047642)

[(C) Fluid Exudation from the Wound 6](#_Toc175047643)

[(E) Wound Bleeding 7](#_Toc175047644)

[(F) Wound Tearing 8](#_Toc175047645)

[(G) Keeping the Wound Dry 8](#_Toc175047646)

[(H) Infection Prevention 9](#_Toc175047647)

[Common Questions: 9](#_Toc175047648)

[止痛药物使用风险 13](#_Toc175047649)

[阿片类药物过度使用是什么？ 13](#_Toc175047650)

[止痛药物的阶梯性选择治疗： 13](#_Toc175047651)

[解热镇痛药物： 13](#_Toc175047652)

[弱阿片类药物： 13](#_Toc175047653)

[强阿片类型的药物： 13](#_Toc175047654)

[Risks of Pain Medication Use 14](#_Toc175047655)

[What is opioid abuse? 14](#_Toc175047656)

[Stepwise selection of pain medication: 14](#_Toc175047657)

[Antipyretic and analgesic medications: 14](#_Toc175047658)

[Weak opioids: 14](#_Toc175047659)

[Strong opioids: 15](#_Toc175047660)

[Common addictive pain medications: 15](#_Toc175047661)

**包皮切割缝合器手术后的维护**

**术后护理手册**

包皮切割缝合术虽然展于微创手术，但是惠者在术后可能会出现伤口轻微疼痛、水肿、血肿、液体渗出、出血等症状。那么，患者在出现上述症状时应如何处理？术后护理时又应把握哪些要点？针对患者的疑问，对术后各种症状的出现及处理方法做出较详细的描达，并对常见问题进行了解回答，希望给医生和患者在术后护理提供及时有效帮助。

（一）术后常见症状的处理

1.术后疼痛

手术麻醉效果消失后，伤口明显有轻微的疼痛，但几小时后疼痛会逐渐减轻、缓解。严

重时可口服镇痛药（以醋氯芬酸等）缓解疼痛。纱布和伤口粘连在一起，下包时易出现撕致、出血、疼痛症状。为了减轻下包疼痛，思者在包扎时最好先用凡士林油纱或凡士林胶帖在切口上包扎，如果没有在下包前，在家用碘伏浸泡伤口部位5-10分钟，会降低粘连程度，从而达到减轻下包疼痛、减少伤口损伤的目的。

2.水肿的原因

包皮切割手术会破坏原有的淋巴循环、微循环，导致大量淋巴液、组织液渗透到包皮内

出现淤积、水肿现象。因此任何手术方式的包皮环切术都会出现不同程度的水肿。包皮切割缝合器在做手术时只要学握好内外板切割的正确位置和尺寸，内板和系带不能留得过长，在8mm左右最佳，一般不会产生水肿现象，因为缝合器做手术的优点在于二个钉之间有空隙，会让淋巴液、组织液渗透出切口到外面。

3.消肿方法

- 术后水肿：一般不用采取任何措施，水肿会自行消退，非常严重时可到医院采取“穿刺引流”消肿。
- 水肿严重可采用高渗盐水或热敷消肿。每天2-3次，每次3-5分钟。高渗盐水由15-30

克食盐加入150毫升温开水制成。

4.水肿消退时间

- 水肿程度和恢复时间与患者自身的淋巴循环和微循环的再造能力及其相关。再造功能强的患者恢复快，再造功能差的患者水肿消退慢。一般水肿周期约一周左右，部分患者水肿时间较长。

（二）血肿怎么会产生的？怎么样处理血肿？

- 血肿的原因是在手术中环切后不能完全的封团缝合导致部分小血管没有钉住或卡住，在不注意的情况下拉动或者特别是拉长阴茎导致血管回缩所产生内出血、血肿。
- 预防血肿的产生， 在整个手术过程中应在自然状态下探作完成，不能在切割后拉长阴茎，如有血肿严重可先挤出淤血然后加压血肿处1--2分钟放手看是否停止出血，如果停止，第一次包扎须紧一点加压包扎8小时至次日，留有淤血一般在一周左右会慢慢退化，如果严重应回医院做检查处理。

（三）伤口液体渗出

- 伤口愈合周期会渗出液体，其中白色分泌物是淋巴液，黄色的是组织液。仅仅渗出白色液体并不是脓液。在伤口出现红、肿、痛、发热等情况下，渗出白色液体应考虑感染发生，患者可去医院检查处理。
- 清洗渗出物的重要性

伤口分泌物会影响肉芽组织生长，不利于伤口愈合。及时清洗伤口渗出物能促进愈合。

- 儿童包茎患者分泌物处理方法

包茎患者分离粘连过程会造成包皮或龟头部分损伤。愈合过程中可能会出现较多的分泌导致愈合周期延长或者感染。患者每天用稀释的碘伏棉球洗去分泌物，再涂抹湿润烧伤膏，或红霉素软膏。

(四)伤口出血

- 术后加压包扎8小时后一般不会出血，晨勃、运动、摩擦和下包时会造成伤口不适等症状是正常的，若伤口出血，用棉签轻压一会即可止血，出血不止时需到医院检查治疗。

(五)伤口撕裂

- 手术十天内不能自行把缝钉垫圈和缝合钉拆掉，如淋浴、运动、摩擦易造成伤口裂开。患者可采用碘伏浸泡的方式进行伤口护理，通常伤口一周内能愈合。若伤口过深、过大可到医院就诊处理。
- 术后护理要点

术后第二天左右应回院做检查，在手术当时没有把缝钉垫圈剪断的同时用尖头眼科剪刀在缝钉垫圈上每隔开2~3颗钉把缝钉垫圈剪断是关键，起到方便脱钉，不易产生水肿及留下影痕。

- 清洗伤口

保持伤口清洁是促进患者术后伤口愈合的要点之一。患者用稀释碘伏（0.5%碘伏自行兑水成0.1%左右）浸泡清洗伤口，或用棉签清洗伤口，每天早晚一次约十天左右。

（六）保持伤口干燥

- 术后伤口渗出的分泌液及残留的尿液使得伤口处于浸溃状态，湿润伤口环境不利于伤口警盒。因此，保持包皮创伤的干燥对伤口愈合时极为重要的，患者可采取以下方法保持伤口的干燥。
- 手术后伤口一直使用消毒过的纸巾包裹约一周即可，利用纸巾的吸附性吸附伤口分泌液和尿液。
- 每天用稀释碘伏清洗伤口2次以上
- 淋浴时采用安全套套在龟头上保护切口不要浸泡在不卫生的水里及伤清洗后必须用电
- 吹风吹干伤口。
- 下包后，尽量穿宽松短裤利于通风，保持伤口干燥。
- 术后分泌液渗出经常会污染内裤，勤换内裤有利于保持干燥环境。

(七)预防感染

每天坚持用碘伏浸泡或清洗伤口时预防感染的重要手段。浸泡或清洗次数越多，效果越好。

常见问题：

1.包扎多长时间为佳？

答:5~7天即可

2.术后能洗澡吗?

答:术后能淋浴但需用安全套要保护切口，但是淋浴后必须用碘伏浸泡伤口并吹干做宽松包扎保护切口

3.小便后尿液浸湿伤口怎么办?

答:小便后用纸巾摺千余尿，保持伤口干燥是利于伤口愈合的基本条件

4.多少天伤口才能愈合?

答:一般在8天左右伤口能愈合。

5.怎样减少勃起?

答:术后应避免受到性刺激，如黄色书籍、录像等。晨勃时小便可解除勃起状态。平时可以专注于其他事情，或听音乐等转移注意力。

6.缝合钉自行脱落算正常吗?

答:缝合钉在10天左右慢慢会排异是正常的。

7.在15~20天左右大部分的患者应能自行脱完，还有部分患者应个体差异所致缝钉未能完全脱落如何处理?

答:如有患者不愿意等下去可去医院进行人工全部拆掉。

8.下包后为什么要穿宽松裤?

答:宽松裤的透气性好，利于伤口干燥，促进伤口愈合。

9.术后饮食有什么禁忌?

答:术后一周内忌酒、腥辣等刺激性食物。多食用鱼、肉、蛋等食品，利于伤口愈合。

10.术后什么时候能同房?

答:缝钉完全自行脱落一周后，为防止伤口裂口。早起同房使用避孕套。

11.术后能否参加体育活动?

答:愈合期间应尽可能减少活动，禁止强烈运动。

Postoperative Care after Circumcision

Although circumcision with suturing is considered a minimally invasive procedure, patients may experience mild postoperative symptoms, such as slight pain at the wound site, edema, hematoma, fluid exudation, and bleeding. How should patients manage these symptoms if they arise? What key points should be noted during the postoperative care? This detailed explanation addresses patients' concerns regarding the appearance of various symptoms after surgery and their management with the aim of providing timely and effective assistance to both doctors and patients in their postoperative care.

(A) Management of Common Postoperative Symptoms

Postoperative Pain

After anesthesia wears off, patients may experience noticeable mild pain at the incision site. However, the pain gradually decreases and alleviates within a few hours. If the pain is severe, oral analgesics, such as diclofenac, can be administered to relieve discomfort. If the dressing has adhered to the wound, removing it can cause tearing, bleeding, and pain. To minimize pain during dressing changes, it is advisable to apply Vaseline-coated gauze or Vaseline before dressing the incision. If this is not done beforehand, soaking the wound area in povidone-iodine for 5-10 minutes at home may reduce adhesion and thereby lessen pain and minimize damage to the wound.

Causes of Edema

Circumcision can disrupt the original lymphatic and microcirculation, leading to the accumulation of a large amount of lymph and tissue fluid in the foreskin, resulting in edema. Therefore, varying degrees of edema can occur with any circumcision technique. If the circumcision device is used properly, ensuring the correct positions and sizes of the inner and outer blades with the inner blade and frenulum not being too long (approximately 8mm is best), edema may not arise. The advantage of using a stapler for circumcision is that there is a gap between the two staples, allowing lymphatic and tissue fluid to seep out through the incision.

Methods to Reduce Swelling

- - **Postoperative Edema**: Generally, no measures are needed as edema will subside on its own. In severe cases, patients should seek hospital treatment for “aspiration drainage” to reduce swelling.
  - For significant edema, hypertonic saline or warm compresses can be applied to alleviate the swelling. This should be done 2-3 times a day for 3-5 minutes each time. Hypertonic saline can be prepared by adding 15-30 grams of salt to 150 milliliters of warm water.

Time for Edema to Subside

The degree and duration of edema are related to the patient's lymphatic circulation and capacity for microcirculation reconstruction. Patients with a strong regenerative ability tend to recover quickly, while those with weaker regenerative capacity may experience prolonged edema. Generally, the period of edema lasts about a week, although some patients may experience it for a longer time.

(B) How Do Hematomas Form? How to Handle Hematomas?

- Hematomas occur when, during surgery, the circumcision site cannot be completely sutured, leading to some small blood vessels being left unsealed. Unintentionally pulling or especially stretching the penis can cause vasoconstriction, resulting in internal bleeding and hematoma.
- To prevent hematoma formation, the surgical procedure should be done in a natural state without overextending the penis after the incision. If a hematoma appears severe, the blood should be expressed, and pressure should be applied to the hematoma for 1-2 minutes to see if bleeding stops. If it does, the initial dressing should be applied tightly and pressed for 8 hours until the next day. Any residual blood is generally absorbed slowly over about a week; however, if the situation is serious, patients should return to the hospital for evaluation and treatment.

(C) Fluid Exudation from the Wound

During the healing period, the wound may exude fluid. The white discharge is lymphatic fluid, while the yellow discharge is tissue fluid. Exuding only white fluid does not indicate purulence. If the wound exhibits redness, swelling, pain, heat, or other symptoms, the presence of white fluid should raise concerns about possible infection, and patients should consult a hospital for evaluation and management.

- **Importance of Cleaning Exudate**:

Wound secretions can impede the growth of granulation tissue and hinder wound healing. Timely cleaning of wound exudate can promote healing.

- **Treatment of Secretions in Pediatric Phimosis Patients**:

The process of separating adhesions in phimosis patients can cause damage to the foreskin or glans. During healing, an excessive amount of secretions may prolong the healing period or lead to infection. Patients should use diluted povidone-iodine on cotton balls to clean the exudate daily, followed by applying a moist burn ointment or erythromycin ointment.

(E) Wound Bleeding

Generally, there should be no bleeding after 8 hours of surgical compression dressing. Symptoms such as morning erections, exercise, friction, and discomfort during dressing changes are normal. If bleeding occurs from the wound, applying gentle pressure with a cotton swab can help stop it. If the bleeding does not stop, a visit to the hospital for examination and treatment is necessary.

(F) Wound Tearing

Do not remove the sutures or staples within ten days post-surgery. Activities such as showering, exercising, or friction can lead to the wound reopening. Patients can care for the wound by soaking it in iodophor; typically, the wound can heal within a week. If the wound is too deep or large, a visit to the hospital for treatment is advised.

Postoperative Care Points

Patients should return to the hospital for a check-up about two days after surgery. It is crucial to use pointed ophthalmic scissors to cut the suture pads, making a cut every 2-3 staples, without cutting the sutures themselves at the time of surgery. This facilitates easier suture removal while reducing the risk of swelling and scarring.

- Cleaning the Wound

Keeping the wound clean is one of the key factors in promoting healing. Patients should clean the wound by soaking it in diluted iodophor (diluting 0.5% iodophor to about 0.1% with water) or using a cotton swab, once in the morning and once in the evening for about ten days.

(G) Keeping the Wound Dry

- The secretion from the postoperative wound and residual urine can cause the wound to become macerated, which is not conducive to healing. Therefore, it is extremely important to keep the wound dry. Patients can adopt the following methods to maintain dryness:
- Keep the wound wrapped with sterile tissue for about a week after surgery to absorb any secretion and urine.
- Clean the wound with diluted iodophor at least twice a day.
- Use a condom during showers to protect the incision from unsanitary water, and ensure that the wound is dried with a hairdryer after washing.
- After dressing changes, wear loose-fitting shorts to promote ventilation and keep the wound dry.
- Surgical secretion often contaminates underwear; frequent changes of underwear help maintain a dry environment.

(H) Infection Prevention

Daily soaking or cleaning of the wound with iodophor is an important measure to prevent infection. The more frequently the soaking or cleaning is done, the better the outcome.

Common Questions:

How long should the dressing stay on?

**Answer:** 5 to 7 days is sufficient.

Can I take a shower after the surgery?

**Answer:** You can take a shower after the surgery, but you need to use a condom to protect the incision. After showering, you must soak the wound in iodophor and dry it with a hairdryer, then apply a loose dressing to protect the incision.

What should I do if urine dampens the wound after urination?

**Answer:** After urinating, use a tissue to wipe off excess urine. Keeping the wound dry is a fundamental condition for promoting healing.

How many days does it take for the wound to heal?

Answer: Generally, the wound can heal in about 8 days.

How can I reduce erections?

**Answer:** After surgery, avoid sexual stimulation, such as reading adult material or watching explicit videos. Urinating during morning erections can help relieve the situation. You can also focus on other activities or listen to music to divert your attention.

Is it normal for the sutures to fall off on their own?

**Answer:** It is normal for the sutures to gradually be rejected around 10 days post-surgery.

Most patients should be able to have their sutures fall off by 15 to 20 days. For some patients, individual differences may cause sutures to not fall off completely. What should be done?

**Answer:** If a patient does not wish to wait, they may go to the hospital to have the sutures removed manually.

Why should I wear loose pants after the surgery?

**Answer:** Loose pants have good breathability, which helps keep the wound dry and promotes healing.

Are there any dietary restrictions after surgery?

**Answer:** Avoid alcohol and spicy or irritating foods within the first week post-surgery. It is beneficial to consume fish, meat, eggs, and other nourishing foods to aid in wound healing.

When can I resume sexual intercourse after the surgery?

**Answer:** It is advisable to wait until the sutures have completely fallen off for about a week to avoid the risk of wound opening. If intercourse occurs earlier, a condom should be used.

Can I participate in physical activities after the surgery?

**Answer:** During the healing period, it is advisable to minimize activity and avoid intense exercise.

止痛药物使用风险

阿片类药物过度使用是什么？

世界范围内，阿片类过度使用致死是毒品相关死亡的主要原因。阿片类药物具有成瘾性，临床上阿片类药物用于疼痛程度重的止痛。当患者因某次损伤或者术后短期内规律服用阿片类药物止痛时，更容易发展为长期使用阿片类的受害者。

止痛药物的阶梯性选择治疗：

取决于疼痛程度，临床止痛需要遵守阶梯性使用原则即：

解热镇痛类→弱阿片类→强阿片类

解热镇痛药物：

该类药物一般不具有成瘾性，但止痛效果相对有限。如【为了避免不必要的商业纷争，我们删除了补充材料当中的具体药物类型】，使用时需要到医院进行检查，排除心脏的疾病，肾脏的疾病，或消化道的疾病，减少不良反应发生。

弱阿片类药物：

1. 如【为了避免不必要的商业纷争，我们删除了补充材料当中的具体药物类型】。

强阿片类型的药物：

1. **用于解热镇痛及弱阿片类药物治疗效果不好时，如【为了避免不必要的商业纷争，我们删除了补充材料当中的具体药物类型】**

常见的成瘾性的止痛药物：（请注意鉴别药品别名）

弱阿片类：【为了避免不必要的商业纷争，我们删除了补充材料当中的具体药物类型】

强阿片类【为了避免不必要的商业纷争，我们删除了补充材料当中的具体药物类型】

Risks of Pain Medication Use

What is opioid abuse?

Globally, opioid overuse is a leading cause of drug-related deaths. Opioid medications are addictive and are used clinically for severe pain management. When patients regularly use opioids for pain relief shortly after an injury or surgery, they are more likely to become long-term opioid users.

Stepwise selection of pain medication:

The choice of pain medication depends on the severity of pain. Clinical pain management should follow a stepwise approach, which is:
**Antipyretic and analgesic medications → Weak opioids → Strong opioids**

Antipyretic and analgesic medications:

This category generally does not have addictive properties, but its pain relief effect is relatively limited. When using these medications, it is necessary to undergo medical examinations to rule out heart, kidney, or gastrointestinal diseases to minimize adverse reactions.

Weak opioids:

Examples include [specific drug types removed to avoid unnecessary commercial disputes].

Strong opioids:

These are used when antipyretic analgesics and weak opioids are ineffective, such as [specific drug types removed to avoid unnecessary commercial disputes].

Common addictive pain medications:

(Please note the importance of identifying the trade names)

- Weak opioids: [specific drug types removed to avoid unnecessary commercial disputes]
- Strong opioids: [specific drug types removed to avoid unnecessary commercial disputes]
