# Supplementary material for: Application of Narrative and AI-Assisted Follow-Up After Voluntary Medical Male Circumcision: Multicenter, Double-Blind, Prospective, Randomized Controlled Trial
Source: J Med Internet Res. 2025 Nov 17;27:e68573. doi: 10.2196/68573 (PMC12622909; doi:10.2196/68573)
Supplement: Multimedia Appendix 2 [file jmir-v27-e68573-s002.docx]

**Table S1.** **Results of repeated measures ANOVA, Sphericity test, Bonferroni test, and Simple effect test for GAD-7, PSQI, ma-NRS, and QoL scores.**

| repeated measures ANOVA | | | | | | | | | | | | | | | | | | | | | | |
| --- | --- | --- | --- | --- | --- | --- | --- | --- | --- | --- | --- | --- | --- | --- | --- | --- | --- | --- | --- | --- | --- | --- |
|  | Sum of Squares SS (Effect) | | | Sum of Squares SS (Error) | | | *df* (Effect) | | *df* (Error) | Mean Square (Effect) | | Mean Square (Error) | | | *F* | *p* | | ges(Generalized eta-squared) | | | | Partial η^2^ |
| GAD-7 |  | | |  | | |  | |  |  | |  | | |  |  | |  | | | |  |
| (Intercept) | 33392.425 | | | 1641.355 | | | 1 | | 386 | 33392.425 | | 4.252 | | | 7852.949 | 0.000 | | 0.872 | | | | 0.953 |
| Group | 273.810 | | | 1641.355 | | | 1 | | 386 | 273.810 | | 4.252 | | | 64.392 | 0.000 | | 0.053 | | | | 0.143 |
| Time | 7774.801 | | | 3259.259 | | | 2 | | 772 | 3887.400 | | 4.222 | | | 920.784 | 0.000 | | 0.613 | | | | 0.705 |
| Group:Time | 536.447 | | | 3259.259 | | | 2 | | 772 | 268.223 | | 4.222 | | | 63.532 | 0.000 | | 0.099 | | | | 0.141 |
| PSQI |  | | |  | | |  | |  |  | |  | | |  |  | |  | | | |  |
| (Intercept) | 60994.772 | | | 3248.810 | | | 1 | | 386 | 60994.772 | | 8.417 | | | 7246.955 | 0.000 | | 0.903 | | | | 0.949 |
| Group | 32.710 | | | 3248.810 | | | 1 | | 386 | 32.710 | | 8.417 | | | 3.886 | 0.049 | | 0.005 | | | | 0.010 |
| Time | 11628.790 | | | 3319.869 | | | 1 | | 386 | 11628.790 | | 8.601 | | | 1352.075 | 0.000 | | 0.639 | | | | 0.778 |
| Group:Time | 48.192 | | | 3319.869 | | | 1 | | 386 | 48.192 | | 8.601 | | | 5.603 | 0.018 | | 0.007 | | | | 0.014 |
| ma-NRS |  | | |  | | |  | |  |  | |  | | |  |  | |  | | | |  |
| (Intercept) | 18075.412 | | | 884.859 | | | 1 | | 386 | 18075.412 | | 2.292 | | | 7884.998 | 0.000 | | 0.856 | | | | 0.953 |
| Group | 0.072 | | | 884.859 | | | 1 | | 386 | 0.072 | | 2.292 | | | 0.031 | 0.860 | | 0.000 | | | | 0.000 |
| Time | 9432.189 | | | 2149.463 | | | 3 | | 1158 | 3144.063 | | 1.856 | | | 1693.830 | 0.000 | | 0.757 | | | | 0.814 |
| Group:Time | 31.695 | | | 2149.463 | | | 3 | | 1158 | 10.565 | | 1.856 | | | 5.692 | 0.001 | | 0.010 | | | | 0.015 |
| Qol |  | | |  | | |  | |  |  | |  | | |  |  | |  | | | |  |
| (Intercept) | 633.854 | | | 1.562 | | | 1 | | 386 | 633.854 | | 0.004 | | | 156653.053 | 0.000 | | 0.995 | | | | 0.998 |
| Group | 0.049 | | | 1.562 | | | 1 | | 386 | 0.049 | | 0.004 | | | 12.145 | 0.001 | | 0.016 | | | | 0.031 |
| Time | 1.739 | | | 1.520 | | | 1 | | 386 | 1.739 | | 0.004 | | | 441.730 | 0.000 | | 0.361 | | | | 0.534 |
| Group:Time | 0.042 | | | 1.520 | | | 1 | | 386 | 0.042 | | 0.004 | | | 10.621 | 0.001 | | 0.013 | | | | 0.027 |
| Sphericity test | | | | | | | | | | | | | | | | | | | | | | |
| GAD-7 | |  | | | | | |  | | | | | |  | | | | | |  | | |
| Within-group effect | | Sphericity W value | | | | | | Sphericity test *p*-value | | | | | | Greenhouse-Geisser (GG) | | | | | | Huynh-Feldt (HF) | | |
| Time | | 0.550 | | | | | | 0.000 | | | | | | 0.690 | | | | | | 0.691 | | |
| Group:Time | | 0.550 | | | | | | 0.000 | | | | | | 0.690 | | | | | | 0.691 | | |
| ma-NRS | |  | | | | | |  | | | | | |  | | | | | |  | | |
| Within-group effect | | Sphericity W value | | | | | | Sphericity test *p*-value | | | | | | Greenhouse-Geisser (GG) | | | | | | Huynh-Feldt (HF) | | |
| Time | | 0.722 | | | | | | 0.000 | | | | | | 0.862 | | | | | | 0.868 | | |
| Group:Time | | 0.722 | | | | | | 0.000 | | | | | | 0.862 | | | | | | 0.868 | | |
| Post-hoc Multiple Comparisons (Time) | | | | | | | | | | | | | | | | | | | | | | |
|  | | | | | MD | | | | | | SE | | | | | | *t* | | | | *p* | |
| GAD-7 | | | | |  | | | | | |  | | | | | |  | | | |  | |
| T0 - T3 | | | | | -6.332 | | | | | | 0.167 | | | | | | -37.936 | | | | 0.000 | |
| T0 - T4 | | | | | -3.227 | | | | | | 0.085 | | | | | | -37.943 | | | | 0.000 | |
| T3 - T4 | | | | | 3.105 | | | | | | 0.174 | | | | | | 17.858 | | | | 0.000 | |
| PSQI | | | | |  | | | | | |  | | | | | |  | | | |  | |
| T0 - T1 | | | | | -7.745 | | | | | | 0.211 | | | | | | -36.771 | | | | 0.000 | |
| ma-NRS | | | | |  | | | | | |  | | | | | |  | | | |  | |
| T1 - T2 | | | | | 2.239 | | | | | | 0.114 | | | | | | 19.584 | | | | 0.000 | |
| T1 - T3 | | | | | 4.553 | | | | | | 0.108 | | | | | | 42.254 | | | | 0.000 | |
| T1 - T4 | | | | | 6.578 | | | | | | 0.083 | | | | | | 79.080 | | | | 0.000 | |
| T2 - T3 | | | | | 2.314 | | | | | | 0.109 | | | | | | 21.198 | | | | 0.000 | |
| T2 - T4 | | | | | 4.339 | | | | | | 0.088 | | | | | | 49.188 | | | | 0.000 | |
| T3 - T4 | | | | | 2.025 | | | | | | 0.078 | | | | | | 25.839 | | | | 0.000 | |
| Qol | | | | |  | | | | | |  | | | | | |  | | | |  | |
| T0 - T4 | | | | | 0.095 | | | | | | 0.005 | | | | | | 21.017 | | | | 0.000 | |
| Simple effects (Time & Group) | | | | | | | | | | | | | | | | | | | | | | |
| GAD-7 | | |  | | |  | | | | | | |  | | | | | |  | | |  |
| Time | | | Group | | | MD | | | | | | | SE | | | | | | *t* | | | *p* |
| T0 | | | SRT vs. NET | | | -0.099 | | | | | | | 0.106 | | | | | | -0.942 | | | 0.347 |
| T3 | | | SRT vs. NET | | | 2.887 | | | | | | | 0.318 | | | | | | 9.079 | | | 0.000 |
| T4 | | | SRT vs. NET | | | 0.124 | | | | | | | 0.137 | | | | | | 0.903 | | | 0.367 |
| PSQI | | |  | | |  | | | | | | |  | | | | | |  | | |  |
| T0 | | | SRT vs. NET | | | -0.088 | | | | | | | 0.213 | | | | | | -0.412 | | | 0.680 |
| T1 | | | SRT vs. NET | | | 0.909 | | | | | | | 0.361 | | | | | | 2.520 | | | 0.012 |
| ma-NRS | | |  | | |  | | | | | | |  | | | | | |  | | |  |
| T1 | | | SRT vs. NET | | | 0.307 | | | | | | | 0.162 | | | | | | 1.900 | | | 0.058 |
| T2 | | | SRT vs. NET | | | -0.453 | | | | | | | 0.168 | | | | | | -2.696 | | | 0.007 |
| T3 | | | SRT vs. NET | | | 0.165 | | | | | | | 0.153 | | | | | | 1.077 | | | 0.282 |
| T4 | | | SRT vs. NET | | | 0.035 | | | | | | | 0.057 | | | | | | 0.611 | | | 0.542 |
| Qol | | |  | | |  | | | | | | |  | | | | | |  | | |  |
| T0 | | | SRT vs. NET | | | -0.001 | | | | | | | 0.003 | | | | | | -0.417 | | | 0.677 |
| T4 | | | SRT vs. NET | | | -0.031 | | | | | | | 0.009 | | | | | | -3.568 | | | 0.000 |

Abbreviation: df, degrees of freedom; ges, generalized eta-squared; MD, mean difference；SE, standard error; GAD, generalized anxiety disorder; PSQI, Pittsburgh sleep quality index; Qol, quality of life; ma-NRS, multiple tools assisted numerical rating scale.

**Table S2. Comparison of GAD-7, PSQI, QoL, and NRS scores and complication rates between the SRT and NET groups post-VMMC.**

| **Variables** | **Total (n = 388)** | **SRT (n = 189)** | **NET (n = 199)** | **Statistic** | ***P*** |
| --- | --- | --- | --- | --- | --- |
|  |  |  |  |  |  |
| **GAD-7(T3), Mean ± SD** | 8.47 ± 3.44 | 9.95 ± 3.50 | 7.06 ± 2.73 | t=9.02 | <0.001 |
| **GAD-7(T4), Mean ± SD** | 5.40 ± 1.35 | 5.46 ± 1.32 | 5.34 ± 1.38 | t=0.90 | 0.367 |
| **PSQI(T4), Mean ± SD** | 12.73 ± 3.58 | 13.20 ± 3.54 | 12.29 ± 3.57 | t=2.52 | 0.012 |
| **Qol(T4), Mean ± SD** | 0.86 ± 0.09 | 0.84 ± 0.09 | 0.87 ± 0.07 | t=-3.55 | <0.001 |
| **NRS(T1), Mean ± SD** | 6.75 ± 1.60 | 6.91 ± 1.49 | 6.60 ± 1.68 | t=1.91 | 0.057 |
| **NRS(T2), Mean ± SD** | 4.52 ± 1.67 | 4.29 ± 1.80 | 4.74 ± 1.50 | t=-2.68 | 0.008 |
| **NRS(T3), Mean ± SD** | 2.20 ± 1.51 | 2.29 ± 1.62 | 2.12 ± 1.40 | t=1.08 | 0.282 |
| **NRS(T4), Mean ± SD** | 0.18 ± 0.56 | 0.20 ± 0.63 | 0.16 ± 0.49 | t=0.61 | 0.542 |
| **Hematoma(T1), n(%)** | 385 (99.23) | 188 (99.47) | 197 (98.99) | χ²=0.00 | 1.000 |
| **Hematoma(T2), n(%)** | 388(100) | 189(100) | 199(100) | - | - |
| **Hematoma(T3), n(%)** | 218 (56.19) | 109 (57.67) | 109 (54.77) | χ²=0.33 | 0.565 |
| **Hematoma(T4), n(%)** | 51 (13.14) | 27 (14.29) | 24 (12.06) | χ²=0.42 | 0.517 |
| **Swelling(T1), n(%)** | 308 (79.38) | 156 (82.54) | 152 (76.38) | χ²=2.25 | 0.134 |
| **Swelling(T2), n(%)** | 345 (88.92) | 165 (87.30) | 180 (90.45) | χ²=0.98 | 0.323 |
| **Swelling(T3), n(%)** | 218 (56.19) | 108 (57.14) | 110 (55.28) | χ²=0.14 | 0.711 |
| **Swelling(T4), n(%)** | 22 (5.67) | 14 (7.41) | 8 (4.02) | χ²=2.08 | 0.149 |
| **Infection(T1),n(%)** | 0(0) | 0(0) | 0(0) | - | - |
| **Infection(T2),n(%)** | 0(0) | 0(0) | 0(0) | - | - |
| **Infection(T3),n(%)** | 4 (1.03) | 2 (1.06) | 2 (1.01) | χ²=0.00 | 1.000 |
| **Infection(T4),n(%)** | 1 (0.26) | 1 (0.53) | 0 (0.00) | - | 0.487 |

Abbreviations: VMMC, Voluntary Medical Male Circumcision; SRT, standardized risk tool; NET, narrative enhanced tool; GAD, generalized anxiety disorder; PSQI, Pittsburgh sleep quality index; Qol, quality of life; SD, standard deviation.

**Table S3. Results of Univariate Regression Analysis of Factors Influencing GAD-7(T3) Scores**

|  | **Beta** | **95%CI** | ***P*** | **R^2^** | **F** |
| --- | --- | --- | --- | --- | --- |
| **Group** | -0.419 | -3.510 ~ -2.264 | 0.000** | 0.176 | F (1,386)=82.431 |
| **Age** | -0.030 | -0.123 ~ 0.067 | 0.562 | 0.001 | F (1,386)=0.337 |
| **BMI** | -0.013 | -0.109 ~ 0.084 | 0.798 | 0.000 | F (1,386)=0.066 |
| **Smoke** | -0.093 | -1.389 ~ 0.043 | 0.066 | 0.009 | F (1,386)=3.393 |
| **Alq** | -0.056 | -1.175 ~ 0.333 | 0.275 | 0.003 | F (1,386)=1.197 |
| **Edu** | 0.002 | -0.441 ~ 0.458 | 0.970 | 0.000 | F (1,386)=0.001 |
| **GAD-7 T0** | -0.010 | -0.363 ~ 0.299 | 0.851 | 0.000 | F (1,386)=0.035 |
| **PSQI T0** | -0.055 | -0.254 ~ 0.074 | 0.283 | 0.003 | F (1,386)=1.156 |
| **QoL T0** | -0.020 | -14.159 ~ 9.444 | 0.696 | 0.000 | F (1,386)=0.153 |
| **Pain T1** | 0.059 | -0.088 ~ 0.341 | 0.249 | 0.003 | F (1,386)=1.332 |
| **Pain T2** | -0.072 | -0.355 ~ 0.056 | 0.155 | 0.005 | F (1,386)=2.031 |
| **Pain T3** | 0.140 | 0.095 ~ 0.546 | 0.006** | 0.020 | F (1,386)=7.765 |
| **Hematoma T1** | 0.063 | -1.424 ~ 6.395 | 0.213 | 0.004 | F (1,386)=1.553 |
| **Hematoma T3** | 0.345 | 1.741 ~ 3.039 | 0.000** | 0.119 | F (1,386)=52.076 |
| **Swelling T1** | 0.017 | -0.701 ~ 0.995 | 0.735 | 0.000 | F (1,386)=0.115 |
| **Swelling T2** | 0.005 | -1.039 ~ 1.147 | 0.923 | 0.000 | F (1,386)=0.009 |
| **Swelling T3** | 0.529 | 3.081 ~ 4.254 | 0.000** | 0.280 | F (1,386)=150.012 |
| **Infection T3** | 0.008 | -3.111 ~ 3.683 | 0.869 | 0.000 | F (1,386)=0.027 |
| **Satisfaction score of analgesic** | -0.142 | -0.969 ~ -0.146 | 0.008** | 0.020 | F (1,340)=7.043 |
| **Analgesic medication used** | 0.120 | 0.179 ~ 2.715 | 0.026* | 0.015 | F (1,340)=5.003 |
| **Opioid medication used** | 0.120 | 0.179 ~ 2.715 | 0.026* | 0.015 | F (1,340)=5.003 |
| **Regularity of analgesic** | -0.193 | -2.309 ~ -0.687 | 0.000** | 0.037 | F (1,340)=13.102 |
| **ORT recall** | -0.166 | -3.132 ~ -0.800 | 0.001** | 0.027 | F (1,386)=10.914 |
| **Follow-up method** | -0.010 | -0.851 ~ 0.694 | 0.842 | 0.000 | F (1,386)=0.040 |
| **Follow-up satisfaction score** | 0.050 | -0.175 ~ 0.525 | 0.328 | 0.002 | F (1,386)=0.959 |

Abbreviations: BMI, body mass index; GAD, generalized anxiety disorder; PSQI, Pittsburgh sleep quality index; Qol, quality of life; CI, confident interval; ORT, opioid risk tool.

**Table S4. Subgroup Analysis of Differences in Post-VMMC Pain Management Between the SRT and NET Groups by Education Level**

| **Variables** | **n (%)** | **SRT** | **NET** | **OR (95%CI)** | ***P*** | **P for interaction** |
| --- | --- | --- | --- | --- | --- | --- |
| **Regularity of analgesic** | | | | | | |
| **All patients** | 388 (100.00) | 100/189 | 154/199 | 5.59 (3.17 ~ 9.86) | <0.001 |  |
| **Education level** |  |  |  |  |  | 0.837 |
| **Primary school graduate or less** | 2 (0.52) | 1/2 | 0/0 |  |  |  |
| **Junior school graduate** | 22 (5.67) | 6/7 | 13/15 | 142422571.37 (0.00 ~ Inf) | 0.992 |  |
| **High school graduate** | 126 (32.47) | 28/53 | 54/73 | 5.06 (1.99 ~ 12.88) | <0.001 |  |
| **College graduate** | 197 (50.77) | 52/103 | 73/94 | 5.33 (2.44 ~ 11.66) | <0.001 |  |
| **Postgraduate or above** | 41 (10.57) | 13/24 | 14/17 | 8.62 (0.94 ~ 78.67) | 0.062 |  |
| **Analgesic medication used** | | | | | | |
| **All patients** | 388 (100.00) | 169/189 | 173/199 | 0.79 (0.42 ~ 1.46) | 0.454 |  |
| **Education level** |  |  |  |  |  | 0.422 |
| **Primary school graduate or less** | 2 (0.52) | 2/2 | 0/0 |  |  |  |
| **Junior school graduate** | 22 (5.67) | 7/7 | 13/15 | 0.00 (0.00 ~ Inf) | 0.996 |  |
| **High school graduate** | 126 (32.47) | 49/53 | 62/73 | 0.46 (0.14 ~ 1.53) | 0.206 |  |
| **College graduate** | 197 (50.77) | 90/103 | 83/94 | 1.09 (0.46 ~ 2.57) | 0.843 |  |
| **Postgraduate or above** | 41 (10.57) | 21/24 | 15/17 | 1.07 (0.16 ~ 7.22) | 0.944 |  |
| **Opioid medication used** | | | | | | |
| **All patients** | 342 (100.00) | 25/169 | 5/173 | 0.17 (0.06 ~ 0.46) | <0.001 |  |
| **Education level** |  |  |  |  |  | 0.254 |
| **Primary school graduate or less** | 2 (0.58) | 0/2 | 0/0 |  |  |  |
| **Junior school graduate** | 20 (5.85) | 0/7 | 0/13 | 1.00 (0.00 ~ Inf) | 1.004 |  |
| **High school graduate** | 111 (32.46) | 9/49 | 0/62 | 0.00 (0.00 ~ Inf) | 0.994 |  |
| **College graduate** | 173 (50.58) | 15/90 | 5/83 | 0.32 (0.11 ~ 0.93) | 0.042 |  |
| **Postgraduate or above** | 36 (10.53) | 1/21 | 0/15 | 0.00 (0.00 ~ Inf) | 0.993 |  |
| **ORT recall** | | | | | | |
| **All patients** | 388 (100.00) | 166/189 | 186/199 | 1.98 (0.97 ~ 4.04) | 0.057 |  |
| **Education level** |  |  |  |  |  | 0.192 |
| **Primary school graduate or less** | 2 (0.52) | 1/2 | 0/0 |  |  |  |
| **Junior school graduate** | 22 (5.67) | 5/7 | 15/15 | 341814171.34 (0.00 ~ Inf) | 0.993 |  |
| **High school graduate** | 126 (32.47) | 50/53 | 68/73 | 0.82 (0.19 ~ 3.57) | 0.792 |  |
| **College graduate** | 197 (50.77) | 91/103 | 88/94 | 1.93 (0.70 ~ 5.38) | 0.211 |  |
| **Postgraduate or above** | 41 (10.57) | 19/24 | 15/17 | 1.97 (0.33 ~ 11.63) | 0.451 |  |
| **Satisfaction score of analgesic** | | | | | | |
| **All patients** | 388 (100.00) | 3.76 ± 0.97 | 4.21 ± 0.70 | 0.46 (0.28 ~ 0.63) * | <0.001 |  |
| **Education level** |  |  |  |  |  | 0.76 |
| **Primary school graduate or less** | 2 (0.52) | 4.50 ± 0.71 | NaN ± NA |  |  |  |
| **Junior school graduate** | 22 (5.67) | 3.57 ± 1.13 | 4.23 ± 0.73 | 0.66 (-0.15 ~ 1.47) | 0.132 |  |
| **High school graduate** | 126 (32.47) | 3.80 ± 1.00 | 4.29 ± 0.66 | 0.49 (0.18 ~ 0.80) | <0.005 |  |
| **College graduate** | 197 (50.77) | 3.68 ± 0.97 | 4.16 ± 0.69 | 0.48 (0.23 ~ 0.73) | <0.001 |  |
| **Postgraduate or above** | 41 (10.57) | 4.00 ± 0.84 | 4.20 ± 0.86 | 0.20 (-0.36 ~ 0.76) | 0.493 |  |

*Data were presented by β (95% CI).

Abbreviations: VMMC, Voluntary Medical Male Circumcision; SRT, standardized risk tool; NET, narrative enhanced tool; OR, Odds Ratio; ORT, Opioid Risk Tool.

**Table S5. Analysis of the mediating effect of education level on postoperative pain management following VMMC in the SRT and NET groups.**

| **Effect** | **Estimate** | **Lower** | **Upper** | **β (95%CI)** | ***P*** | **Mediation** |
| --- | --- | --- | --- | --- | --- | --- |
| **Regularity of analgesic** | | | | | | |
| **Indirect** | 0.00 | -0.00 | 0.01 | 0.00 (-0.00 ~ 0.01) | 0.640 | 0.40 |
| **Direct** | 0.30 | 0.20 | 0.37 | 0.30 (0.20 ~ 0.37) | <0.001 | 99.60 |
| **Total** | 0.30 | 0.20 | 0.37 | 0.30 (0.20 ~ 0.37) | <0.001 | 100.00 |
| **Analgesic medication used** | | | | | | |
| **Indirect** | 0.00 | -0.00 | 0.01 | 0.00 (-0.00 ~ 0.01) | 0.640 | -2.73 |
| **Direct** | -0.02 | -0.08 | 0.04 | -0.02 (-0.08 ~ 0.04) | 0.520 | 102.73 |
| **Total** | -0.02 | -0.07 | 0.04 | -0.02 (-0.07 ~ 0.04) | 0.560 | 100.00 |
| **Opioid medication used** | | | | | | |
| **Indirect** | -0.00 | -0.01 | 0.01 | -0.00 (-0.01 ~ 0.01) | 0.840 | 0.31 |
| **Direct** | -0.12 | -0.19 | -0.06 | -0.12 (-0.19 ~ -0.06) | <0.001 | 99.69 |
| **Total** | -0.12 | -0.20 | -0.06 | -0.12 (-0.20 ~ -0.06) | <0.001 | 100.00 |
| **ORT recall** | | | | | | |
| **Indirect** | 0.00 | -0.00 | 0.01 | 0.00 (-0.00 ~ 0.01) | 0.400 | 4.17 |
| **Direct** | 0.06 | 0.00 | 0.10 | 0.06 (0.00 ~ 0.10) | 0.080 | 95.83 |
| **Total** | 0.06 | 0.00 | 0.11 | 0.06 (0.00 ~ 0.11) | <0.001 | 100.00 |
| **Satisfaction score of analgesic** | | | | | | |
| **Indirect** | 0.00 | -0.01 | 0.02 | 0.00 (-0.01 ~ 0.02) | 0.680 | 0.42 |
| **Direct** | 0.47 | 0.28 | 0.63 | 0.47 (0.28 ~ 0.63) | <0.001 | 99.58 |
| **Total** | 0.47 | 0.29 | 0.63 | 0.47 (0.29 ~ 0.63) | <0.001 | 100.00 |

Abbreviations: VMMC, Voluntary Medical Male Circumcision; SRT, standardized risk tool; NET, narrative enhanced tool; OR, Odds Ratio; ORT, Opioid Risk Tool.

P for the indirect effect > 0.05 suggests that education level does not indirectly influence NET’s direct effect on pain management.

**Table S6.** **Mediator path analysis of education level on postoperative pain management following VMMC in the SRT and NET groups.**

| **Path** | **Relation** | **β** | **SE** | **Lower** | **Upper** | ***P*** | **β (95%CI)** |
| --- | --- | --- | --- | --- | --- | --- | --- |
| **Regularity of analgesic** | | | | | | | |
| **NET → Education level** | **Exposure→ Mediator** | -0.14 | 0.08 | -0.30 | 0.02 | 0.098 | -0.14 (-0.30 ~ 0.02) |
| **NET → Regularity of analgesic** | **Exposure→ Outcome** | 1.71 | 0.29 | 1.14 | 2.28 | <.001 | 1.71 (1.14 ~ 2.28) |
| **Education level→ Regularity of analgesic** | **Mediator→ Outcome** | -0.08 | 0.17 | -0.41 | 0.26 | 0.656 | -0.08 (-0.41 ~ 0.26 |
| **Analgesic medication used** | | | | | | | |
| **NET→Education level** | **Exposure→ Mediator** | -0.17 | 0.08 | -0.32 | -0.02 | 0.026 | -0.17 (-0.32 ~ -0.02) |
| **NET→Analgesic medication used** | **Exposure→ Outcome** | -0.26 | 0.32 | -0.88 | 0.37 | 0.419 | -0.26 (-0.88 ~ 0.37) |
| **Education level→Analgesic medication used** | **Mediator→ Outcome** | -0.11 | 0.21 | -0.52 | 0.31 | 0.616 | -0.11 (-0.52 ~ 0.31) |
| **Opioid medication used** | | | | | | | |
| **NET→Education level** | **Exposure→ Mediator** | -0.14 | 0.08 | -0.30 | 0.02 | 0.098 | -0.14 (-0.30 ~ 0.02) |
| **NET→Opioid medication used** | **Exposure→ Outcome** | -1.75 | 0.50 | -2.74 | -0.77 | <0.001 | -1.75 (-2.74 ~ -0.77) |
| **Education level→Opioid medication used** | **Mediator→ Outcome** | 0.08 | 0.26 | -0.42 | 0.58 | 0.760 | 0.08 (-0.42 ~ 0.58) |
| **ORT recall** | | | | | | | |
| **NET→Education level** | **Exposure→ Mediator** | -0.17 | 0.08 | -0.32 | -0.02 | 0.026 | -0.17 (-0.32 ~ -0.02) |
| **NET→ORT recall** | **Exposure→ Outcome** | 0.65 | 0.37 | -0.06 | 1.37 | 0.075 | 0.65 (-0.06 ~ 1.37) |
| **Edu****→ORT recall** | **Mediator→ Outcome** | -0.20 | 0.24 | -0.66 | 0.27 | 0.408 | -0.20 (-0.66 ~ 0.27) |
| **Satisfaction score of analgesic** | | | | | | | |
| **NET→Education level** | **Exposure→ Mediator** | -0.14 | 0.08 | -0.30 | 0.02 | 0.098 | -0.14 (-0.30 ~ 0.02) |
| **NET→Satisfaction score of analgesic** | **Exposure→ Outcome** | 0.45 | 0.09 | 0.27 | 0.63 | <0.001 | 0.45 (0.27 ~ 0.63) |
| **Education level→Satisfaction score of analgesic** | **Mediator→ Outcome** | -0.02 | 0.06 | -0.14 | 0.10 | 0.735 | -0.02 (-0.14 ~ 0.10) |

Abbreviations: VMMC, Voluntary Medical Male Circumcision; SRT, standardized risk tool; NET, narrative enhanced tool; OR, Odds Ratio; ORT, Opioid Risk Tool.

**Table S7. Subgroup Analysis of Differences in GAD-7, PSQI, QoL, and NRS Scores Between SRT and NET Groups After VMMC by Follow-Up Method (TTC/AAC).**

| **Variables** | **n (%)** | **SRT** | **NET** | **β (95%CI)** | ***P*** | **P for interaction** |
| --- | --- | --- | --- | --- | --- | --- |
| **GAD-7(T3)** | | | | | | |
| **All patients** | 388 (100.00) | 9.95 ± 3.50 | 7.06 ± 2.73 | -2.89 (-3.51 ~ -2.26) | <0.001 |  |
| **Follow-up** |  |  |  |  |  | 0.797 |
| **TTC** | **105 (27.06)** | 9.89 ± 3.51 | 7.13 ± 2.63 | -2.75 (-3.94 ~ -1.56) | <0.001 |  |
| **AAC** | **283 (72.94)** | 9.97 ± 3.51 | 7.03 ± 2.77 | -2.94 (-3.67 ~ -2.20) | <0.001 |  |
| **GAD-7(T4)** | | | | | | |
| **All patients** | **388 (100.00)** | 5.46 ± 1.32 | 5.34 ± 1.38 | -0.12 (-0.39 ~ 0.14) | 0.367 |  |
| **Follow-up** |  |  |  |  |  | 0.795 |
| **TTC** | **105 (27.06)** | 5.40 ± 1.28 | 5.21 ± 1.18 | -0.18 (-0.65 ~ 0.29) | 0.443 |  |
| **AAC** | **283 (72.94)** | 5.49 ± 1.34 | 5.38 ± 1.44 | -0.10 (-0.43 ~ 0.22) | 0.529 |  |
| **PSQI(T4)** | | | | | | |
| **All patients** | **388 (100.00)** | 13.20 ± 3.54 | 12.29 ± 3.57 | -0.91 (-1.62 ~ -0.20) | 0.012 |  |
| **Follow-up** |  |  |  |  |  | 0.343 |
| **TTC** | **105 (27.06)** | 12.74 ± 3.87 | 12.38 ± 3.99 | -0.35 (-1.86 ~ 1.15) | 0.648 |  |
| **AAC** | **283 (72.94)** | 13.38 ± 3.40 | 12.25 ± 3.42 | -1.12 (-1.92 ~ -0.33) | 0.006 |  |
| **QoL(T4)** | | | | | | |
| **All patients** | **388 (100.00)** | 0.84 ± 0.09 | 0.87 ± 0.07 | 0.03 (0.01 ~ 0.05) | <0.001 |  |
| **Follow-up** |  |  |  |  |  | 0.335 |
| **TTC** | **105 (27.06)** | 0.83 ± 0.08 | 0.87 ± 0.07 | 0.04 (0.01 ~ 0.07) | 0.004 |  |
| **AAC** | **283 (72.94)** | 0.85 ± 0.10 | 0.87 ± 0.08 | 0.03 (0.01 ~ 0.05) | 0.015 |  |
| **NRS(T1)** | | | | | | |
| **All patients** | **388 (100.00)** | 6.91 ± 1.49 | 6.60 ± 1.68 | -0.31 (-0.62 ~ 0.01) | 0.058 |  |
| **Follow-up** |  |  |  |  |  | 0.227 |
| **TTC** | **105 (27.06)** | 6.77 ± 1.51 | 6.79 ± 1.77 | 0.01 (-0.62 ~ 0.65) | 0.963 |  |
| **AAC** | **283 (72.94)** | 6.96 ± 1.48 | 6.54 ± 1.65 | -0.43 (-0.79 ~ -0.06) | 0.023 |  |
| **NRS(T2)** | | | | | | |
| **All patients** | **388 (100.00)** | 4.29 ± 1.80 | 4.74 ± 1.50 | 0.45 (0.12 ~ 0.78) | 0.007 |  |
| **Follow-up** |  |  |  |  |  | 0.342 |
| **TTC** | **105 (27.06)** | 4.11 ± 1.77 | 4.83 ± 1.61 | 0.71 (0.07 ~ 1.36) | 0.033 |  |
| **AAC** | **283 (72.94)** | 4.36 ± 1.82 | 4.71 ± 1.46 | 0.35 (-0.03 ~ 0.74) | 0.071 |  |
| **NRS(T3)** | | | | | | |
| **All patients** | **388 (100.00)** | 2.29 ± 1.62 | 2.12 ± 1.40 | -0.17 (-0.47 ~ 0.14) | 0.282 |  |
| **Follow-up** |  |  |  |  |  | 0.080 |
| **TTC** | **105 (27.06)** | 2.51 ± 1.65 | 1.90 ± 1.47 | -0.61 (-1.20 ~ -0.01) | 0.050 |  |
| **AAC** | **283 (72.94)** | 2.20 ± 1.60 | 2.20 ± 1.37 | -0.00 (-0.35 ~ 0.35) | 0.994 |  |
| **NRS(T4)** | | | | | | |
| **All patients** | **388 (100.00)** | 0.20 ± 0.63 | 0.16 ± 0.49 | -0.03 (-0.15 ~ 0.08) | 0.542 |  |
| **Follow-up** |  |  |  |  |  | 0.548 |
| **TTC** | **105 (27.06)** | 0.19 ± 0.62 | 0.10 ± 0.30 | -0.09 (-0.28 ~ 0.09) | 0.335 |  |
| **AAC** | **283 (72.94)** | 0.20 ± 0.64 | 0.18 ± 0.54 | -0.01 (-0.15 ~ 0.12) | 0.832 |  |

Abbreviations: VMMC, Voluntary Medical Male Circumcision; SRT, standardized risk tool; NET, narrative enhanced tool; GAD, generalized anxiety disorder; PSQI, Pittsburgh sleep quality index; Qol, quality of life; AAC, AI-assisted consulting; TTC, two-way texting consulting.

All adjusted by education level and age.

**Table S8. Analysis of the Mediating Effect of AAC on the Impact of NET on GAD-7, PSQI, QoL, and NRS Scores Post-VMMC.**

| **Effect** | **Estimate** | **Lower** | **Upper** | **β (95%CI)** | ***P*** | **Mediation** |  |
| --- | --- | --- | --- | --- | --- | --- | --- |
|  |  |  |  |  |  |  |  |
| **GAD-7(T3)** | | | | | | |  |
| **Indirect** | -0.00 | -0.04 | 0.02 | -0.00 (-0.04 ~ 0.02) | 0.880 | -0.02 |  |
| **Direct** | -2.96 | -3.46 | -2.41 | -2.96 (-3.46 ~ -2.41) | <0.001 | 100.02 |  |
| **Total** | -2.96 | -3.44 | -2.40 | -2.96 (-3.44 ~ -2.40) | <0.001 | 100.00 |  |
| **GAD-7(T4)** | | | | | | |  |
| **Indirect** | 0.00 | -0.01 | 0.02 | 0.00 (-0.01 ~ 0.02) | 0.800 | -0.38 |  |
| **Direct** | -0.17 | -0.38 | 0.07 | -0.17 (-0.38 ~ 0.07) | 0.240 | 100.38 |  |
| **Total** | -0.16 | -0.36 | 0.07 | -0.16 (-0.36 ~ 0.07) | 0.240 | 100.00 |  |
| **PSQI(T4)** | | | | | | |  |
| **Indirect** | 0.00 | -0.05 | 0.03 | 0.00 (-0.05 ~ 0.03) | 0.920 | -0.04 |  |
| **Direct** | -1.07 | -1.64 | -0.45 | -1.07 (-1.64 ~ -0.45) | <0.001 | 100.04 |  |
| **Total** | -1.07 | -1.59 | -0.45 | -1.07 (-1.59 ~ -0.45) | <0.001 | 100.00 |  |
| **QoL(T4)** | | | | | | |  |
| **Indirect** | 0.00 | -0.00 | 0.00 | 0.00 (-0.00 ~ 0.00) | 0.760 | 0.36 |  |
| **Direct** | 0.03 | 0.02 | 0.05 | 0.03 (0.02 ~ 0.05) | <0.001 | 99.64 |  |
| **Total** | 0.03 | 0.02 | 0.05 | 0.03 (0.02 ~ 0.05) | <0.001 | 100.00 |  |
| **NRS(T1)** | | | | | | |  |
| **Indirect** | -0.00 | -0.02 | 0.01 | -0.00 (-0.02 ~ 0.01) | 0.960 | 0.01 |  |
| **Direct** | -0.32 | -0.58 | -0.04 | -0.32 (-0.58 ~ -0.04) | <0.001 | 99.99 |  |
| **Total** | -0.32 | -0.57 | -0.04 | -0.32 (-0.57 ~ -0.04) | <0.001 | 100.00 |  |
| **NRS(T2)** | | | | | | |  |
| **Indirect** | 0.00 | -0.02 | 0.01 | 0.00 (-0.02 ~ 0.01) | 0.880 | 0.10 |  |
| **Direct** | 0.43 | 0.16 | 0.72 | 0.43 (0.16 ~ 0.72) | <0.001 | 99.90 |  |
| **Total** | 0.43 | 0.18 | 0.72 | 0.43 (0.18 ~ 0.72) | <0.001 | 100.00 |  |
| **NRS(T3)** | | | | | | |  |
| **Indirect** | -0.00 | -0.02 | 0.01 | -0.00(-0.02 ~ 0.01) | 0.920 | 0.04 |  |
| **Direct** | -0.19 | -0.44 | 0.07 | -0.19 (-0.44 ~ 0.07) | 0.200 | 99.96 |  |
| **Total** | -0.19 | -0.42 | 0.07 | -0.19 (-0.42 ~ 0.07) | 0.240 | 100.00 |  |
| **NRS(T4)** | | | | | | |  |
| **Indirect** | 0.00 | -0.01 | 0.01 | 0.00 (-0.01 ~ 0.01) | 0.800 | -0.21 |  |
| **Direct** | -0.05 | -0.14 | 0.05 | -0.05 (-0.14 ~ 0.05) | 0.240 | 100.21 |  |
| **Total** | -0.05 | -0.13 | 0.05 | -0.05 (-0.13 ~ 0.05) | 0.360 | 100.00 |  |

Abbreviations: VMMC, Voluntary Medical Male Circumcision; SRT, standardized risk tool; NET, narrative enhanced tool; AAC, AI-assisted consulting; GAD, generalized anxiety disorder; PSQI, Pittsburgh sleep quality index; Qol, quality of life; NRS, numerical rating scale.

P for the indirect effect > 0.05 suggests that AAC does not indirectly influence NET’s direct effect on GAD-7, PSQI, QoL, or NRS levels.

All adjusted by education level and age.

**Table S9. Mediator path analysis of AAC on GAD-7/PSQI/QoL/NRS scores following VMMC in the SRT and NET groups.**

| **Path** | **Relation** | **β** | **SE** | **Lower** | **Upper** | ***P*** | **β (95%CI)** |
| --- | --- | --- | --- | --- | --- | --- | --- |
| **GAD-7(T3)** | | | | | | | |
| **NET → AAC** | **Exposure→ Mediator** | 0.08 | 0.23 | -0.37 | 0.54 | 0.720 | 0.08 (-0.37 ~ 0.54) |
| **Group → GAD-7(T3)** | **Exposure→ Outcome** | -2.91 | 0.32 | -3.55 | -2.28 | <0.001 | -2.91 (-3.55 ~ -2.28) |
| **AAC→ GAD-7(T3)** | **Mediator→ Outcome** | -0.01 | 0.36 | -0.72 | 0.70 | 0.986 | -0.01 (-0.72 ~ 0.70) |
| **GAD-7(T4)** | | | | | | | |
| **NET → AAC** | **Exposure→ Mediator** | 0.08 | 0.23 | -0.37 | 0.54 | 0.720 | 0.08 (-0.37 ~ 0.54) |
| **Group → GAD-7(T4)** | **Exposure→ Outcome** | -0.15 | 0.14 | -0.42 | 0.13 | 0.294 | -0.15 (-0.42 ~ 0.13) |
| **AAC→ GAD-7(T4)** | **Mediator→ Outcome** | 0.12 | 0.16 | -0.18 | 0.43 | 0.424 | 0.12 (-0.18 ~ 0.43) |
| **PSQI(T4)** | | | | | | | |
| **NET → AAC** | **Exposure→ Mediator** | 0.08 | 0.23 | -0.37 | 0.54 | 0.720 | 0.08 (-0.37 ~ 0.54) |
| **Group → PSQI(T4)** | **Exposure→ Outcome** | -1.02 | 0.36 | -1.73 | -0.31 | 0.005 | -1.02 (-1.73 ~ -0.31) |
| **AAC→ PSQI(T4)** | **Mediator→ Outcome** | 0.15 | 0.41 | -0.64 | 0.95 | 0.705 | 0.15 (-0.64 ~ 0.95) |
| **QoL(T4)** | | | | | | | |
| **NET → AAC** | **Exposure→ Mediator** | 0.08 | 0.23 | -0.37 | 0.54 | 0.720 | 0.08 (-0.37 ~ 0.54) |
| **Group →QoL(T4)** | **Exposure→ Outcome** | 0.03 | 0.01 | 0.02 | 0.05 | <0.001 | 0.03 (0.02 ~ 0.05) |
| **AAC→ QoL(T4)** | **Mediator→ Outcome** | 0.01 | 0.01 | -0.01 | 0.03 | 0.318 | 0.01 (-0.01 ~ 0.03) |
| **NRS(T1)** | | | | | | | |
| **NET → AAC** | **Exposure→ Mediator** | 0.08 | 0.23 | -0.37 | 0.54 | 0.720 | 0.08 (-0.37 ~ 0.54) |
| **Group →NRS(T1)** | **Exposure→ Outcome** | -0.30 | 0.16 | -0.62 | 0.02 | 0.069 | -0.30 (-0.62 ~ 0.02) |
| **AAC→ NRS(T1)** | **Mediator→ Outcome** | -0.03 | 0.18 | -0.39 | 0.33 | 0.883 | -0.03 (-0.39 ~ 0.33) |
| **NRS(T2)** | | | | | | | |
| **NET → AAC** | **Exposure→ Mediator** | 0.08 | 0.23 | -0.37 | 0.54 | 0.720 | 0.08 (-0.37 ~ 0.54) |
| **Group →NRS(T2)** | **Exposure→ Outcome** | 0.45 | 0.17 | 0.12 | 0.79 | 0.009 | 0.45 (0.12 ~ 0.79) |
| **AAC→ NRS(T2)** | **Mediator→ Outcome** | 0.06 | 0.19 | -0.31 | 0.44 | 0.742 | 0.06 (-0.31 ~ 0.44) |
| **NRS(T3)** | | | | | | | |
| **NET → AAC** | **Exposure→ Mediator** | 0.08 | 0.23 | -0.37 | 0.54 | 0.720 | 0.08 (-0.37 ~ 0.54) |
| **Group →NRS(T3)** | **Exposure→ Outcome** | -0.17 | 0.16 | -0.48 | 0.13 | 0.271 | -0.17 (-0.48 ~ 0.13) |
| **AAC→ NRS(T3)** | **Mediator→ Outcome** | 0.03 | 0.17 | -0.32 | 0.37 | 0.884 | 0.03 (-0.32 ~ 0.37) |
| **NRS(T4)** | | | | | | | |
| **NET → AAC** | **Exposure→ Mediator** | 0.08 | 0.23 | -0.37 | 0.54 | 0.720 | 0.08 (-0.37 ~ 0.54) |
| **Group →NRS(T4)** | **Exposure→ Outcome** | -0.04 | 0.06 | -0.16 | 0.07 | 0.440 | -0.04 (-0.16 ~ 0.07) |
| **AAC→ NRS(T4)** | **Mediator→ Outcome** | 0.05 | 0.06 | -0.08 | 0.18 | 0.427 | 0.05 (-0.08 ~ 0.18) |

Abbreviations: VMMC, Voluntary Medical Male Circumcision; SRT, standardized risk tool; NET, narrative enhanced tool; AAC, AI-assisted consulting; GAD, generalized anxiety disorder; PSQI, Pittsburgh sleep quality index; Qol, quality of life; NRS, numerical rating scale.

All adjusted by education level and age.

**Table S10.** **Examples of Patient Consultation Text Samples Using the AAC Platform**

| **A：**  **[Input]** What is the reason for my ongoing swelling?  **[Output]** Hello, [patient's name]! After circumcision, penile swelling is a relatively common phenomenon and usually improves within a few days after the surgery. Currently, your titanium clips have completely fallen off, but you may still experience postoperative swelling. The swelling at 21 days post-surgery may be related to the tissue healing process. However, the degree and duration of swelling can vary among individuals.  I recommend you consider the following points:  Observe Changes: If the swelling has not significantly worsened and is not accompanied by other symptoms (such as noticeable pain, redness, swelling, or fever), there is usually no need to worry too much.  Keep the Area Clean and Dry: Ensure that the surgical area remains clean to avoid infection, which could exacerbate the swelling.  Rest Appropriately: During this time, try to avoid strenuous activities to give your body more time to heal.  Diet and Fluid Intake: Maintain a balanced diet, drink plenty of water, and reduce salt intake, as this can help alleviate swelling.  Follow-Up: If the swelling persists or if you experience any other discomfort, it is advisable to return to the hospital for a follow-up. The doctor can assess your condition and provide appropriate recommendations. Please click here to contact the healthcare staff [Link…].  Please consider your feelings and symptoms to determine the best course of action. I wish you a speedy recovery! | Discussion:  The intelligent agent extracts previous conversation memories to identify the patient's current postoperative day. At the same time, the information that the patient's titanium clips have completely fallen off has been registered and uploaded by the doctor in the backend, thus enabling a more individualized care approach for the patient. |
| --- | --- |
| **B：**  **[Input]** I am very worried about the wound healing and the possibility of infection. It's difficult for me to fall asleep, and I'm feeling very anxious.  **[Output]** Hello, [patient's name]! I understand your current feelings very well. It's natural to feel worried after surgery. Your anxiety and unease are understandable because during the recovery process, concerns about wound healing and infection are common.  Remember, you are not alone. Many people have similar feelings when going through similar situations. Firstly, it's okay to have some worries as it can help you take better care of yourself and stay vigilant. Pay attention to your body, but also trust in its powerful ability to heal itself.  If you're feeling uneasy, try doing something relaxing, like deep breathing, imagining a peaceful place with your eyes closed, or listening to soothing music. These can help relieve anxiety and promote better sleep.  Remember, maintaining communication with your doctor is important. If you have any symptoms that worry you, seek their help promptly. Trust that during this recovery process, you can gradually adjust and move towards improvement. Some situations take time to heal, so be patient with yourself and believe that things will get better.  I hope you can find inner peace and sleep peacefully soon! If you need to talk about more things or share your feelings, I'm here to listen. | Discussion:  Generative AI tends to use more compassionate phrases. While comforting the patient's emotions, it also provides suggestions for addressing these issues. |
| **C：**  **[Input]** May I ask which antimicrobial medications would be the most effective for me to take right now?  **[Output]** Hello, [patient's name]! If you notice that your wound is indeed experiencing symptoms such as pain, fever, or pus, you may consider taking oral antibiotics to treat the infection. However, please be sure to note that you should not take penicillin antibiotics, as our system has detected that you have a history of penicillin allergy, which could lead to serious adverse reactions. It is recommended that you opt for cephalosporin or quinolone medications for treatment. Wishing you a speedy recovery! If necessary, feel free to visit the hospital for an in-person consultation, and we will be more than happy to assist you. | Discussion:  The patient's medical information, including their medical history and allergy history, is logged into the AI system, allowing for better adjustments when providing relevant recommendations, ensuring the safety of the advice given. |

**Figure S1 Demonstration of follow-up timeline and content for VMMC surgery patients**


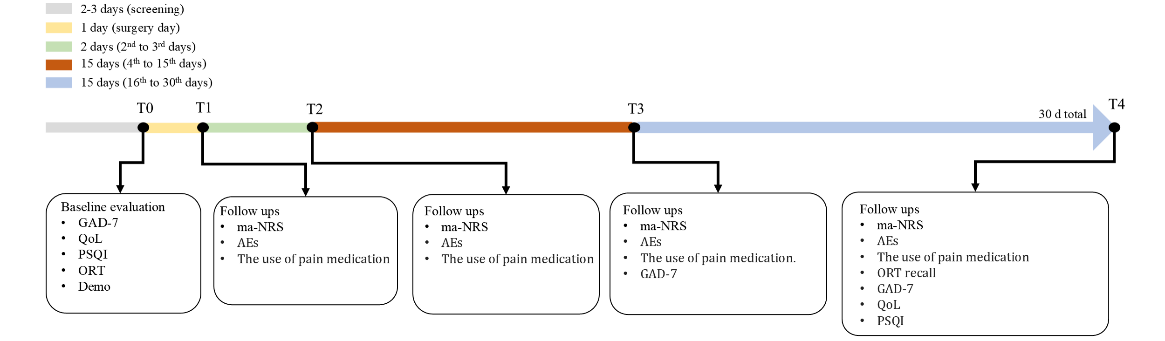


"Surgery day" is recognized as the first day of the trail.

Abbreviations: GAD, generalized anxiety disorder; QoL, quality of life score; PSIQ, Pittsburgh sleep quality index; Demo, demographics data; AE, adverse event; ORT, opioid risk tool.

**Figure S2 Schematic workflow of the AAC platform for postoperative follow-up of VMMC patients**


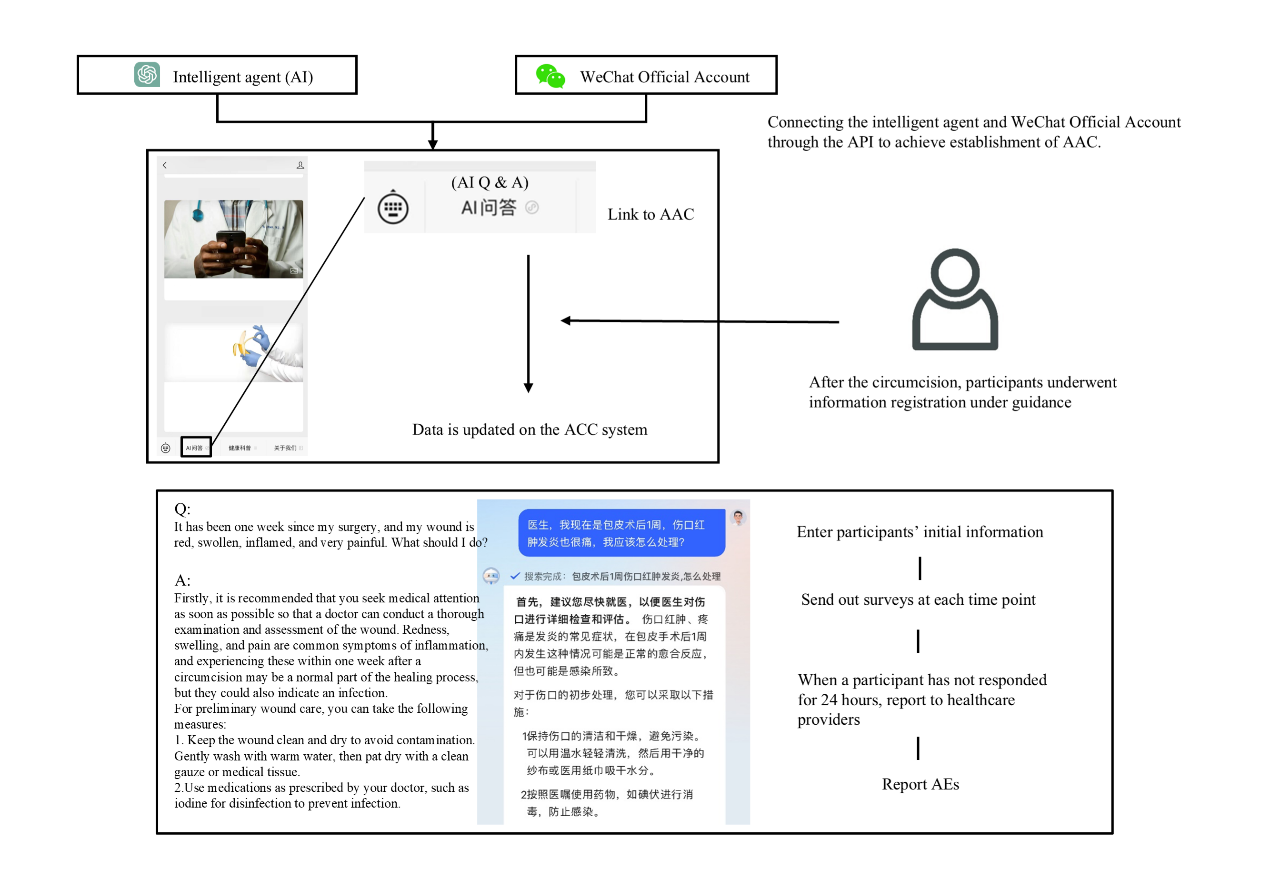


After completing the surgery, participants were asked to wait in the room mentioned earlier for 30 minutes. Investigator would have distributed the manual and guided participants to access AAC through the WeChat public account during this time, as well as recorded participants' names and surgery dates. The WeChat public account could be quickly accessed through WeChat (a widely used SMS platform in China), and then clicking “link to AAC” would redirect to the generative AI dialogue box.

Abbreviations: AAC, AI-assisted consulting; API: application programming interface.
