# Supplementary material for: Application of Narrative and AI-Assisted Follow-Up After Voluntary Medical Male Circumcision: Multicenter, Double-Blind, Prospective, Randomized Controlled Trial
Source: J Med Internet Res. 2025 Nov 17;27:e68573. doi: 10.2196/68573 (PMC12622909; doi:10.2196/68573)
Supplement: Checklist 1 [file jmir-v27-e68573-s003.pdf]

CONSORT-EHEALTH (V 1.6.1) - Submission/Publication Form

The CONSORT-EHEALTH checklist is intended for authors of randomized trials evaluating web-based and Internet-based applications/interventions, including mobile interventions, electronic games (incl multiplayer games), social media, certain telehealth applications, and other interactive and/or networked electronic applications. Some of the items (e.g. all subitems under item 5 - description of the intervention) may also be applicable for other study designs.

The goal of the CONSORT EHEALTH checklist and guideline is to be
a) a guide for reporting for authors of RCTs,
b) to form a basis for appraisal of an ehealth trial (in terms of validity)

CONSORT-EHEALTH items/subitems are MANDATORY reporting items for studies published in the Journal of Medical Internet Research and other journals / scientific societies endorsing the checklist.

Items numbered 1., 2., 3., 4a., 4b etc are original CONSORT or CONSORT-NPT (non-pharmacologic treatment) items.
Items with Roman numerals (i., ii, iii, iv etc.) are CONSORT-EHEALTH extensions/clarifications.

As the CONSORT-EHEALTH checklist is still considered in a formative stage, we would ask that you also RATE ON A SCALE OF 1-5 how important/useful you feel each item is FOR THE PURPOSE OF THE CHECKLIST and reporting guideline (optional).

Mandatory reporting items are marked with a red *.
In the textboxes, either copy & paste the relevant sections from your manuscript into this form - please include any quotes from your manuscript in QUOTATION MARKS,
or answer directly by providing additional information not in the manuscript, or elaborating on why the item was not relevant for this study.

YOUR ANSWERS WILL BE PUBLISHED AS A SUPPLEMENTARY FILE TO YOUR PUBLICATION IN JMIR AND ARE CONSIDERED PART OF YOUR PUBLICATION (IF ACCEPTED).
Please fill in these questions diligently. Information will not be copyedited, so please use proper spelling and grammar, use correct capitalization, and avoid abbreviations.

DO NOT FORGET TO SAVE AS PDF _AND_ CLICK THE SUBMIT BUTTON SO YOUR ANSWERS ARE IN OUR DATABASE !!!

Citation Suggestion (if you append the pdf as Appendix we suggest to cite this paper in the caption):
Eysenbach G, CONSORT-EHEALTH Group
CONSORT-EHEALTH: Improving and Standardizing Evaluation Reports of Web-based and Mobile Health Interventions
J Med Internet Res 2011;13(4):e126

URL: http://www.jmir.org/2011/4/e126/
doi: 10.2196/jmir.1923
PMID: 22209829

urologistwanguro@gmail.com Switch account

Not shared

Draft saved

* Indicates required question

Your name *

First Last

Linfeng Wang

Primary Affiliation (short), City, Country *

University of Toronto, Toronto, Canada

The First Affiliated Hospital of Chongqing Med

Your e-mail address *

abc@gmail.com

urologistwanguro@gmail.com

Title of your manuscript *

Provide the (draft) title of your manuscript.

Application of Narrative and AI-Assisted Follow-Up After Voluntary Medical Male Circumcision: A Multicenter, Double-Blind, Prospective, Randomized Controlled Trial

Name of your App/Software/Intervention *

If there is a short and a long/alternate name, write the short name first and add the long name in brackets.

NET (Narrative Enhenced Tool)

Evaluated Version (if any)

e.g. "V1", "Release 2017-03-01", "Version 2.0.27913"

Your answer

Language(s) *

What language is the intervention/app in? If multiple languages are available, separate by comma (e.g. "English, French")

Chinese

URL of your Intervention Website or App

e.g. a direct link to the mobile app on app in appstore (itunes, Google Play), or URL of the website. If the intervention is a DVD or hardware, you can also link to an Amazon page.

Your answer

URL of an image/screenshot (optional)

Your answer

Accessibility *

Can an enduser access the intervention presently?

- (●) access is free and open
- ( ) access only for special usergroups, not open
- ( ) access is open to everyone, but requires payment/subscription/in-app purchases
- ( ) app/intervention no longer accessible
- ( ) Other:

Primary Medical Indication/Disease/Condition *

e.g. "Stress", "Diabetes", or define the target group in brackets after the condition, e.g. "Autism (Parents of children with)", "Alzheimers (Informal Caregivers of)"

Circumcation

Primary Outcomes measured in trial *

comma-separated list of primary outcomes reported in the trial

Anxiety, Sleep quality, Quality of life, Pain, Drug

Secondary/other outcomes

Are there any other outcomes the intervention is expected to affect?

Your answer

Recommended "Dose" *

What do the instructions for users say on how often the app should be used?

- ◯ Approximately Daily
- ◯ Approximately Weekly
- ◯ Approximately Monthly
- ◯ Approximately Yearly
- ◉ "as needed"
- ◯ Other:

Approx. Percentage of Users (starters) still using the app as recommended after 3 months *

- ◉ unknown / not evaluated
- ◯ 0-10%
- ◯ 11-20%
- ◯ 21-30%
- ◯ 31-40%
- ◯ 41-50%
- ◯ 51-60%
- ◯ 61-70%
- ◯ 71%-80%
- ◯ 81-90%
- ◯ 91-100%
- ◯ Other:

Overall, was the app/intervention effective? *

- (selected) yes: all primary outcomes were significantly better in intervention group vs control
- partly: SOME primary outcomes were significantly better in intervention group vs control
- no statistically significant difference between control and intervention
- potentially harmful: control was significantly better than intervention in one or more outcomes
- inconclusive: more research is needed
- Other:

Article Preparation Status/Stage *

At which stage in your article preparation are you currently (at the time you fill in this form)

- not submitted yet - in early draft status
- not submitted yet - in late draft status, just before submission
- submitted to a journal but not reviewed yet
- (selected) submitted to a journal and after receiving initial reviewer comments
- submitted to a journal and accepted, but not published yet
- published
- Other:

Journal *

If you already know where you will submit this paper (or if it is already submitted), please provide the journal name (if it is not JMIR, provide the journal name under "other")

- ○ not submitted yet / unclear where I will submit this
- ◉ Journal of Medical Internet Research (JMIR)
- ○ JMIR mHealth and UHealth
- ○ JMIR Serious Games
- ○ JMIR Mental Health
- ○ JMIR Public Health
- ○ JMIR Formative Research
- ○ Other JMIR sister journal
- ○ Other:

Is this a full powered effectiveness trial or a pilot/feasibility trial? *

- ◉ Pilot/feasibility
- ○ Fully powered

Manuscript tracking number *

If this is a JMIR submission, please provide the manuscript tracking number under "other" (The ms tracking number can be found in the submission acknowledgement email, or when you login as author in JMIR. If the paper is already published in JMIR, then the ms tracking number is the four-digit number at the end of the DOI, to be found at the bottom of each published article in JMIR)

- ○ no ms number (yet) / not (yet) submitted to / published in JMIR
- ◉ Other: JMIR ms#68573

TITLE AND ABSTRACT

1a) TITLE: Identification as a randomized trial in the title

1a) Does your paper address CONSORT item 1a? *

I.e does the title contain the phrase "Randomized Controlled Trial"? (if not, explain the reason under "other")

- (selected) yes
- ( ) Other:

1a-i) Identify the mode of delivery in the title

Identify the mode of delivery. Preferably use “web-based” and/or “mobile” and/or “electronic game” in the title. Avoid ambiguous terms like “online”, “virtual”, “interactive”. Use “Internet-based” only if Intervention includes non-web-based Internet components (e.g. email), use “computer-based” or “electronic” only if offline products are used. Use “virtual” only in the context of “virtual reality” (3-D worlds). Use “online” only in the context of “online support groups”. Complement or substitute product names with broader terms for the class of products (such as “mobile” or “smart phone” instead of “iphone”), especially if the application runs on different platforms.

	1	2	3	4	5	
subitem not at all important	○	○	●	○	○	essential

Clear selection

Does your paper address subitem 1a-i? *

Copy and paste relevant sections from manuscript title (include quotes in quotation marks "like this" to indicate direct quotes from your manuscript), or elaborate on this item by providing additional information not in the ms, or briefly explain why the item is not applicable/relevant for your study

My manuscript title does not include sub projects 1a-i, as our intervention combines multiple methods.

1a-ii) Non-web-based components or important co-interventions in title

Mention non-web-based components or important co-interventions in title, if any (e.g., "with telephone support").

	1	2	3	4	5	
subitem not at all important	○	○	○	○	○	essential

Does your paper address subitem 1a-ii?

Copy and paste relevant sections from manuscript title (include quotes in quotation marks "like this" to indicate direct quotes from your manuscript), or elaborate on this item by providing additional information not in the ms, or briefly explain why the item is not applicable/relevant for your study

Your answer

1a-iii) Primary condition or target group in the title

Mention primary condition or target group in the title, if any (e.g., "for children with Type I Diabetes") Example: A Web-based and Mobile Intervention with Telephone Support for Children with Type I Diabetes: Randomized Controlled Trial

	1	2	3	4	5	
subitem not at all important	○	○	○	○	◉	essential

Clear selection

Does your paper address subitem 1a-iii? *

Copy and paste relevant sections from manuscript title (include quotes in quotation marks "like this" to indicate direct quotes from your manuscript), or elaborate on this item by providing additional information not in the ms, or briefly explain why the item is not applicable/relevant for your study

My manuscript title includes the project and clearly states that the tool is intended for patients undergoing circumcision.Like "Application of Narrative and AI-Assisted Follow-Up After Voluntary Medical Male Circumcision".

1b) ABSTRACT: Structured summary of trial design, methods, results, and conclusions

NPT extension: Description of experimental treatment, comparator, care providers, centers, and blinding status.

1b-i) Key features/functionalities/components of the intervention and comparator in the METHODS section of the ABSTRACT

Mention key features/functionalities/components of the intervention and comparator in the abstract. If possible, also mention theories and principles used for designing the site. Keep in mind the needs of systematic reviewers and indexers by including important synonyms. (Note: Only report in the abstract what the main paper is reporting. If this information is missing from the main body of text, consider adding it)

	1	2	3	4	5	
subitem not at all important	○	○	○	◉	○	essential

Clear selection

Does your paper address subitem 1b-i? *

Copy and paste relevant sections from the manuscript abstract (include quotes in quotation marks "like this" to indicate direct quotes from your manuscript), or elaborate on this item by providing additional information not in the ms, or briefly explain why the item is not applicable/relevant for your study

“Participants were randomly assigned to standardized risk tool (SRT) or narrative enhanced tool (NET) group. In addition to the regular postoperative communication, NET group received an extra narrative video. AI-assisted consulting (AAC) was introduced to facilitate follow-ups.”

1b-ii) Level of human involvement in the METHODS section of the ABSTRACT

Clarify the level of human involvement in the abstract, e.g., use phrases like “fully automated” vs. “therapist/nurse/care provider/physician-assisted” (mention number and expertise of providers involved, if any). (Note: Only report in the abstract what the main paper is reporting. If this information is missing from the main body of text, consider adding it)

	1	2	3	4	5	
subitem not at all important	○	○	●	○	○	essential

Clear selection

Does your paper address subitem 1b-ii?

Copy and paste relevant sections from the manuscript abstract (include quotes in quotation marks "like this" to indicate direct quotes from your manuscript), or elaborate on this item by providing additional information not in the ms, or briefly explain why the item is not applicable/relevant for your study

Your answer

1b-iii) Open vs. closed, web-based (self-assessment) vs. face-to-face assessments in the METHODS section of the ABSTRACT

Mention how participants were recruited (online vs. offline), e.g., from an open access website or from a clinic or a closed online user group (closed usergroup trial), and clarify if this was a purely web-based trial, or there were face-to-face components (as part of the intervention or for assessment). Clearly say if outcomes were self-assessed through questionnaires (as common in web-based trials). Note: In traditional offline trials, an open trial (open-label trial) is a type of clinical trial in which both the researchers and participants know which treatment is being administered. To avoid confusion, use “blinded” or “unblinded” to indicated the level of blinding instead of “open”, as “open” in web-based trials usually refers to “open access” (i.e. participants can self-enrol). (Note: Only report in the abstract what the main paper is reporting. If this information is missing from the main body of text, consider adding it)

	1	2	3	4	5	
subitem not at all important	○	●	○	○	○	essential

Clear selection

Does your paper address subitem 1b-iii?

Copy and paste relevant sections from the manuscript abstract (include quotes in quotation marks "like this" to indicate direct quotes from your manuscript), or elaborate on this item by providing additional information not in the ms, or briefly explain why the item is not applicable/relevant for your study

Your answer

1b-iv) RESULTS section in abstract must contain use data

Report number of participants enrolled/assessed in each group, the use/uptake of the intervention (e.g., attrition/adherence metrics, use over time, number of logins etc.), in addition to primary/secondary outcomes. (Note: Only report in the abstract what the main paper is reporting. If this information is missing from the main body of text, consider adding it)

	1	2	3	4	5	
subitem not at all important	○	◉	○	○	○	essential

Clear selection

Does your paper address subitem 1b-iv?

Copy and paste relevant sections from the manuscript abstract (include quotes in quotation marks "like this" to indicate direct quotes from your manuscript), or elaborate on this item by providing additional information not in the ms, or briefly explain why the item is not applicable/relevant for your study

Your answer

1b-v) CONCLUSIONS/DISCUSSION in abstract for negative trials

Conclusions/Discussions in abstract for negative trials: Discuss the primary outcome - if the trial is negative (primary outcome not changed), and the intervention was not used, discuss whether negative results are attributable to lack of uptake and discuss reasons. (Note: Only report in the abstract what the main paper is reporting. If this information is missing from the main body of text, consider adding it)

	1	2	3	4	5	
subitem not at all important	○	○	◉	○	○	essential

Clear selection

Does your paper address subitem 1b-v?

Copy and paste relevant sections from the manuscript abstract (include quotes in quotation marks "like this" to indicate direct quotes from your manuscript), or elaborate on this item by providing additional information not in the ms, or briefly explain why the item is not applicable/relevant for your study

Your answer

INTRODUCTION

2a) In INTRODUCTION: Scientific background and explanation of rationale

2a-i) Problem and the type of system/solution

Describe the problem and the type of system/solution that is object of the study: intended as stand-alone intervention vs. incorporated in broader health care program? Intended for a particular patient population? Goals of the intervention, e.g., being more cost-effective to other interventions, replace or complement other solutions? (Note: Details about the intervention are provided in “Methods” under 5)

	1	2	3	4	5	
subitem not at all important	○	○	○	○	◉	essential

Clear selection

Does your paper address subitem 2a-i? *

Copy and paste relevant sections from the manuscript (include quotes in quotation marks "like this" to indicate direct quotes from your manuscript), or elaborate on this item by providing additional information not in the ms, or briefly explain why the item is not applicable/relevant for your study

“To our knowledge, researchers have focused on addressing anxiety during the perioperative period of circumcision through distraction, virtual reality, or music therapy[20-22]. However, few studies have assessed the effects of narrative communication in alleviating anxiety in patients after circumcision. ”

2a-ii) Scientific background, rationale: What is known about the (type of) system

Scientific background, rationale: What is known about the (type of) system that is the object of the study (be sure to discuss the use of similar systems for other conditions/diagnoses, if appropiate), motivation for the study, i.e. what are the reasons for and what is the context for this specific study, from which stakeholder viewpoint is the study performed, potential impact of findings [2]. Briefly justify the choice of the comparator.

	1	2	3	4	5	
subitem not at all important	○	○	○	◉	○	essential

Clear selection

Does your paper address subitem 2a-ii? *

Copy and paste relevant sections from the manuscript (include quotes in quotation marks "like this" to indicate direct quotes from your manuscript), or elaborate on this item by providing additional information not in the ms, or briefly explain why the item is not applicable/relevant for your study

“Katz et al. provided support for the effects of narrative risk communication in influencing feelings of cancer risk through ease of imagination [19]. Collectively, narratives with detailed personal experiences allow listeners to concentrate more on the content[17], which may enhance postoperative patients' awareness of surgical risks.”

2b) In INTRODUCTION: Specific objectives or hypotheses

Does your paper address CONSORT subitem 2b? *

Copy and paste relevant sections from the manuscript (include quotes in quotation marks "like this" to indicate direct quotes from your manuscript), or elaborate on this item by providing additional information not in the ms, or briefly explain why the item is not applicable/relevant for your study

“to evaluate the effectiveness of narrative-enhanced communication by assessing the anxiety scores of the participants. Additionally, we introduced an agent-based AI-assisted follow-up tool to assess its value for the first time in a trial.”

METHODS

3a) Description of trial design (such as parallel, factorial) including allocation ratio

Does your paper address CONSORT subitem 3a? *

Copy and paste relevant sections from the manuscript (include quotes in quotation marks "like this" to indicate direct quotes from your manuscript), or elaborate on this item by providing additional information not in the ms, or briefly explain why the item is not applicable/relevant for your study

“This multicenter, double-blind, randomized clinical trial was conducted in five urology departments in Chongqing, China. The trial was approved by the Ethics Committee of the First Affiliated Hospital of Chongqing Medical University (2023-264) and followed the principles of the Declaration of Helsinki. The trial was conducted from July 1, 2023, to April 29, 2024, and has been registered in the Clinical Trial Registry (No. ChiCTR2300076099). This study followed the Consolidated Standards of Reporting Trials (CONSORT) reporting guidelines[23]. Written informed consent from themselves or guardians was obtained from all participants.”

3b) Important changes to methods after trial commencement (such as eligibility criteria), with reasons

Does your paper address CONSORT subitem 3b? *

Copy and paste relevant sections from the manuscript (include quotes in quotation marks "like this" to indicate direct quotes from your manuscript), or elaborate on this item by providing additional information not in the ms, or briefly explain why the item is not applicable/relevant for your study

“Follow-ups were conducted at the corresponding time points after circumcision (Figure S1). In the early stages of the trial, we regularly sent online survey questionnaires to participants via the two-way texting consulting (TTC) and conducted online dialogue with participants. From the midpoint of the trial, follow-up was conducted with the assistance of the AI. We constructed a TTC platform based on short message service (SMS) to provide patients with more efficient telemedicine services. Researchers have indicated that AI algorithms can potentially be applied to perform tedious tasks that are time-consuming and devoid of intellectual challenges[24]. In this study, we also recognized this and introduced "intelligent agents" to optimize the follow-up procedure from October 10, 2023.”

3b-i) Bug fixes, Downtimes, Content Changes

Bug fixes, Downtimes, Content Changes: ehealth systems are often dynamic systems. A description of changes to methods therefore also includes important changes made on the intervention or comparator during the trial (e.g., major bug fixes or changes in the functionality or content) (5-iii) and other “unexpected events” that may have influenced study design such as staff changes, system failures/downtimes, etc. [2].

	1	2	3	4	5	
subitem not at all important	○	○	●	○	○	essential

Clear selection

Does your paper address subitem 3b-i?

Copy and paste relevant sections from the manuscript (include quotes in quotation marks "like this" to indicate direct quotes from your manuscript), or elaborate on this item by providing additional information not in the ms, or briefly explain why the item is not applicable/relevant for your study

Your answer

4a) Eligibility criteria for participants

Does your paper address CONSORT subitem 4a? *

Copy and paste relevant sections from the manuscript (include quotes in quotation marks "like this" to indicate direct quotes from your manuscript), or elaborate on this item by providing additional information not in the ms, or briefly explain why the item is not applicable/relevant for your study

“The participants in this research were recruited continuously, and the inclusion criteria included male patients (≥15 years) who received VMMC for phimosis or redundant prepuce and who could use smartphones to complete web-based questionnaires. The exclusion criteria included a history of mental disorders, baseline 7-item Generalized Anxiety Disorder (GAD-7) score ≥ 10, unable to take opioids or non-steroidal anti-inflammatory drugs (NSAIDs) for any reason, and unable to provide written informed consent.”

4a-i) Computer / Internet literacy

Computer / Internet literacy is often an implicit “de facto” eligibility criterion - this should be explicitly clarified.

	1	2	3	4	5	
subitem not at all important	○	○	○	◉	○	essential

Clear selection

Does your paper address subitem 4a-i?

Copy and paste relevant sections from the manuscript (include quotes in quotation marks "like this" to indicate direct quotes from your manuscript), or elaborate on this item by providing additional information not in the ms, or briefly explain why the item is not applicable/relevant for your study

Your answer

4a-ii) Open vs. closed, web-based vs. face-to-face assessments:

Open vs. closed, web-based vs. face-to-face assessments: Mention how participants were recruited (online vs. offline), e.g., from an open access website or from a clinic, and clarify if this was a purely web-based trial, or there were face-to-face components (as part of the intervention or for assessment), i.e., to what degree got the study team to know the participant. In online-only trials, clarify if participants were quasi-anonymous and whether having multiple identities was possible or whether technical or logistical measures (e.g., cookies, email confirmation, phone calls) were used to detect/prevent these.

	1	2	3	4	5	
subitem not at all important	○	○	○	○	◉	essential

Clear selection

Does your paper address subitem 4a-ii? *

Copy and paste relevant sections from the manuscript (include quotes in quotation marks "like this" to indicate direct quotes from your manuscript), or elaborate on this item by providing additional information not in the ms, or briefly explain why the item is not applicable/relevant for your study

“The specific process of the trial included a 2-day screening period (varying by hospital), 1 d for surgery, and a 30-day follow-up period (Figure S1). Before surgery, investigators will take participants into a private meeting room for preoperative communication, which, as required, includes the following: the surgical procedure of circumcision, the purpose and necessity of the surgery, and the potential risks associated with the surgery. At the same time, the investigators will instruct the participants on how to correctly fill out the online questionnaire to ensure validity and reliability. All participants were required to return to the room after the circumcision for 30-minute vital signs monitoring. Meanwhile, investigators would provide different manuals based on the assigned groups. Both manuals provided general guidelines on postoperative recovery and strategies for coping with postoperative complications. ”

4a-iii) Information giving during recruitment

Information given during recruitment. Specify how participants were briefed for recruitment and in the informed consent procedures (e.g., publish the informed consent documentation as appendix, see also item X26), as this information may have an effect on user self-selection, user expectation and may also bias results.

	1	2	3	4	5	
subitem not at all important	○	○	◉	○	○	essential

Clear selection

Does your paper address subitem 4a-iii?

Copy and paste relevant sections from the manuscript (include quotes in quotation marks "like this" to indicate direct quotes from your manuscript), or elaborate on this item by providing additional information not in the ms, or briefly explain why the item is not applicable/relevant for your study

Your answer

4b) Settings and locations where the data were collected

Does your paper address CONSORT subitem 4b? *

Copy and paste relevant sections from the manuscript (include quotes in quotation marks "like this" to indicate direct quotes from your manuscript), or elaborate on this item by providing additional information not in the ms, or briefly explain why the item is not applicable/relevant for your study

This study involves the setting and location of data collection, and the specific content is included in the figure.

4b-i) Report if outcomes were (self-)assessed through online questionnaires

Clearly report if outcomes were (self-)assessed through online questionnaires (as common in web-based trials) or otherwise.

	1	2	3	4	5	
subitem not at all important	○	○	○	○	◉	essential

Clear selection

Does your paper address subitem 4b-i? *

Copy and paste relevant sections from the manuscript (include quotes in quotation marks "like this" to indicate direct quotes from your manuscript), or elaborate on this item by providing additional information not in the ms, or briefly explain why the item is not applicable/relevant for your study

“AI-assisted consulting (AAC) is implemented through an application programming interface (API) that integrates a generative intelligent agent with WeChat”

4b-ii) Report how institutional affiliations are displayed

Report how institutional affiliations are displayed to potential participants [on ehealth media], as affiliations with prestigious hospitals or universities may affect volunteer rates, use, and reactions with regards to an intervention.(Not a required item – describe only if this may bias results)

	1	2	3	4	5	
subitem not at all important	○	○	◉	○	○	essential

Clear selection

Does your paper address subitem 4b-ii?

Copy and paste relevant sections from the manuscript (include quotes in quotation marks "like this" to indicate direct quotes from your manuscript), or elaborate on this item by providing additional information not in the ms, or briefly explain why the item is not applicable/relevant for your study

Your answer

5) The interventions for each group with sufficient details to allow replication, including how and when they were actually administered

5-i) Mention names, credential, affiliations of the developers, sponsors, and owners

Mention names, credential, affiliations of the developers, sponsors, and owners [6] (if authors/evaluators are owners or developer of the software, this needs to be declared in a "Conflict of interest" section or mentioned elsewhere in the manuscript).

	1	2	3	4	5	
subitem not at all important	○	◉	○	○	○	essential

Clear selection

Does your paper address subitem 5-i?

Copy and paste relevant sections from the manuscript (include quotes in quotation marks "like this" to indicate direct quotes from your manuscript), or elaborate on this item by providing additional information not in the ms, or briefly explain why the item is not applicable/relevant for your study

Your answer

5-ii) Describe the history/development process

Describe the history/development process of the application and previous formative evaluations (e.g., focus groups, usability testing), as these will have an impact on adoption/use rates and help with interpreting results.

	1	2	3	4	5	
subitem not at all important	○	◉	○	○	○	essential

Clear selection

Does your paper address subitem 5-ii?

Copy and paste relevant sections from the manuscript (include quotes in quotation marks "like this" to indicate direct quotes from your manuscript), or elaborate on this item by providing additional information not in the ms, or briefly explain why the item is not applicable/relevant for your study

Your answer

?

5-iii) Revisions and updating

Revisions and updating. Clearly mention the date and/or version number of the application/intervention (and comparator, if applicable) evaluated, or describe whether the intervention underwent major changes during the evaluation process, or whether the development and/or content was “frozen” during the trial. Describe dynamic components such as news feeds or changing content which may have an impact on the replicability of the intervention (for unexpected events see item 3b).

	1	2	3	4	5	
subitem not at all important	○	◉	○	○	○	essential

Clear selection

Does your paper address subitem 5-iii?

Copy and paste relevant sections from the manuscript (include quotes in quotation marks "like this" to indicate direct quotes from your manuscript), or elaborate on this item by providing additional information not in the ms, or briefly explain why the item is not applicable/relevant for your study

Your answer

5-iv) Quality assurance methods

Provide information on quality assurance methods to ensure accuracy and quality of information provided [1], if applicable.

	1	2	3	4	5	
subitem not at all important	○	◉	○	○	○	essential

Clear selection

Does your paper address subitem 5-iv?

Copy and paste relevant sections from the manuscript (include quotes in quotation marks "like this" to indicate direct quotes from your manuscript), or elaborate on this item by providing additional information not in the ms, or briefly explain why the item is not applicable/relevant for your study

Your answer

5-v) Ensure replicability by publishing the source code, and/or providing screenshots/screen-capture video, and/or providing flowcharts of the algorithms used

Ensure replicability by publishing the source code, and/or providing screenshots/screen-capture video, and/or providing flowcharts of the algorithms used. Replicability (i.e., other researchers should in principle be able to replicate the study) is a hallmark of scientific reporting.

	1	2	3	4	5	
subitem not at all important	○	○	◉	○	○	essential

Clear selection

Does your paper address subitem 5-v?

Copy and paste relevant sections from the manuscript (include quotes in quotation marks "like this" to indicate direct quotes from your manuscript), or elaborate on this item by providing additional information not in the ms, or briefly explain why the item is not applicable/relevant for your study

Your answer

5-vi) Digital preservation

Digital preservation: Provide the URL of the application, but as the intervention is likely to change or disappear over the course of the years; also make sure the intervention is archived (Internet Archive, webcitation.org, and/or publishing the source code or screenshots/videos alongside the article). As pages behind login screens cannot be archived, consider creating demo pages which are accessible without login.

	1	2	3	4	5	
subitem not at all important	○	○	◉	○	○	essential

Clear selection

Does your paper address subitem 5-vi?

Copy and paste relevant sections from the manuscript (include quotes in quotation marks "like this" to indicate direct quotes from your manuscript), or elaborate on this item by providing additional information not in the ms, or briefly explain why the item is not applicable/relevant for your study

Your answer

5-vii) Access

Access: Describe how participants accessed the application, in what setting/context, if they had to pay (or were paid) or not, whether they had to be a member of specific group. If known, describe how participants obtained "access to the platform and Internet" [1]. To ensure access for editors/reviewers/readers, consider to provide a "backdoor" login account or demo mode for reviewers/readers to explore the application (also important for archiving purposes, see vi).

	1	2	3	4	5	
subitem not at all important	○	○	◉	○	○	essential

Clear selection

Does your paper address subitem 5-vii? *

Copy and paste relevant sections from the manuscript (include quotes in quotation marks "like this" to indicate direct quotes from your manuscript), or elaborate on this item by providing additional information not in the ms, or briefly explain why the item is not applicable/relevant for your study

"A narrative-enhanced audiovisual communication tool was employed. The tool was designed through a combined process, in which our research team demonstrated postoperative risks and information on the use of analgesics and then combined this with video narratives that included real stories from patients with opioid use disorder (OUD). These narratives were filmed by a professional photography team, included the reasons for initial exposure to opioids (including chronic pain). All these narratives were from speakers in China. The combined video had a length of approximately 10 min [Multimedia Appendix 1: Narrative Enhenced Tool_vedio], and the intervention occurred within 30 min post-surgery, with the investigators confirming that the manuals had been thoroughly read."

5-viii) Mode of delivery, features/functionalities/components of the intervention and comparator, and the theoretical framework

Describe mode of delivery, features/functionalities/components of the intervention and comparator, and the theoretical framework [6] used to design them (instructional strategy [1], behaviour change techniques, persuasive features, etc., see e.g., [7, 8] for terminology). This includes an in-depth description of the content (including where it is coming from and who developed it) [1]," whether [and how] it is tailored to individual circumstances and allows users to track their progress and receive feedback" [6]. This also includes a description of communication delivery channels and – if computer-mediated communication is a component – whether communication was synchronous or asynchronous [6]. It also includes information on presentation strategies [1], including page design principles, average amount of text on pages, presence of hyperlinks to other resources, etc. [1].

	1	2	3	4	5	
subitem not at all important	○	○	●	○	○	essential

Clear selection

Does your paper address subitem 5-viii? *

Copy and paste relevant sections from the manuscript (include quotes in quotation marks "like this" to indicate direct quotes from your manuscript), or elaborate on this item by providing additional information not in the ms, or briefly explain why the item is not applicable/relevant for your study

This study involves the setting and location of data collection, and the specific content is included in the main figure.

5-ix) Describe use parameters

Describe use parameters (e.g., intended "doses" and optimal timing for use). Clarify what instructions or recommendations were given to the user, e.g., regarding timing, frequency, heaviness of use, if any, or was the intervention used ad libitum.

	1	2	3	4	5	
subitem not at all important	○	○	○	◉	○	essential

Clear selection

Does your paper address subitem 5-ix?

Copy and paste relevant sections from the manuscript (include quotes in quotation marks "like this" to indicate direct quotes from your manuscript), or elaborate on this item by providing additional information not in the ms, or briefly explain why the item is not applicable/relevant for your study

Your answer

5-x) Clarify the level of human involvement

Clarify the level of human involvement (care providers or health professionals, also technical assistance) in the e-intervention or as co-intervention (detail number and expertise of professionals involved, if any, as well as “type of assistance offered, the timing and frequency of the support, how it is initiated, and the medium by which the assistance is delivered”. It may be necessary to distinguish between the level of human involvement required for the trial, and the level of human involvement required for a routine application outside of a RCT setting (discuss under item 21 – generalizability).

	1	2	3	4	5	
subitem not at all important	○	○	○	○	◉	essential

Clear selection

Does your paper address subitem 5-x?

Copy and paste relevant sections from the manuscript (include quotes in quotation marks "like this" to indicate direct quotes from your manuscript), or elaborate on this item by providing additional information not in the ms, or briefly explain why the item is not applicable/relevant for your study

Your answer

5-xi) Report any prompts/reminders used

Report any prompts/reminders used: Clarify if there were prompts (letters, emails, phone calls, SMS) to use the application, what triggered them, frequency etc. It may be necessary to distinguish between the level of prompts/reminders required for the trial, and the level of prompts/reminders for a routine application outside of a RCT setting (discuss under item 21 – generalizability).

	1	2	3	4	5	
subitem not at all important	○	◉	○	○	○	essential

Clear selection

Does your paper address subitem 5-xi? *

Copy and paste relevant sections from the manuscript (include quotes in quotation marks "like this" to indicate direct quotes from your manuscript), or elaborate on this item by providing additional information not in the ms, or briefly explain why the item is not applicable/relevant for your study

This study involves the use of reminders, and the specific reminder times for participants have been created in the flowchart.

5-xii) Describe any co-interventions (incl. training/support)

Describe any co-interventions (incl. training/support): Clearly state any interventions that are provided in addition to the targeted eHealth intervention, as ehealth intervention may not be designed as stand-alone intervention. This includes training sessions and support [1]. It may be necessary to distinguish between the level of training required for the trial, and the level of training for a routine application outside of a RCT setting (discuss under item 21 – generalizability.

	1	2	3	4	5	
subitem not at all important	○	○	○	◉	○	essential

Clear selection

Does your paper address subitem 5-xii? *

Copy and paste relevant sections from the manuscript (include quotes in quotation marks "like this" to indicate direct quotes from your manuscript), or elaborate on this item by providing additional information not in the ms, or briefly explain why the item is not applicable/relevant for your study

"Before surgery, investigators will take participants into a private meeting room for preoperative communication, which, as required, includes the following: the surgical procedure of circumcision, the purpose and necessity of the surgery, and the potential risks associated with the surgery. At the same time, the investigators will instruct the participants on how to correctly fill out the online questionnaire to ensure validity and reliability. All participants were required to return to the room after the circumcision for 30-minute vital signs monitoring. Meanwhile, investigators would provide different manuals based on the assigned groups. Both manuals provided general guidelines on postoperative recovery and strategies for coping with postoperative complications."

6a) Completely defined pre-specified primary and secondary outcome measures, including how and when they were assessed

Does your paper address CONSORT subitem 6a? *

Copy and paste relevant sections from the manuscript (include quotes in quotation marks "like this" to indicate direct quotes from your manuscript), or elaborate on this item by providing additional information not in the ms, or briefly explain why the item is not applicable/relevant for your study

This study involves pre setting outcome indicators, and the specific evaluation time has been created in the flowchart.

6a-i) Online questionnaires: describe if they were validated for online use and apply CHERRIES items to describe how the questionnaires were designed/deployed

If outcomes were obtained through online questionnaires, describe if they were validated for online use and apply CHERRIES items to describe how the questionnaires were designed/deployed [9].

	1	2	3	4	5	
subitem not at all important	○	○	◉	○	○	essential

Clear selection

Does your paper address subitem 6a-i?

Copy and paste relevant sections from manuscript text

Your answer

6a-ii) Describe whether and how “use” (including intensity of use/dosage) was defined/measured/monitored

Describe whether and how “use” (including intensity of use/dosage) was defined/measured/monitored (logins, logfile analysis, etc.). Use/adoption metrics are important process outcomes that should be reported in any ehealth trial.

	1	2	3	4	5	
subitem not at all important	○	○	◉	○	○	essential

Clear selection

Does your paper address subitem 6a-ii?

Copy and paste relevant sections from manuscript text

Your answer

6a-iii) Describe whether, how, and when qualitative feedback from participants was obtained

Describe whether, how, and when qualitative feedback from participants was obtained (e.g., through emails, feedback forms, interviews, focus groups).

	1	2	3	4	5	
subitem not at all important	○	○	○	◉	○	essential

Clear selection

Does your paper address subitem 6a-iii?

Copy and paste relevant sections from manuscript text

Your answer

6b) Any changes to trial outcomes after the trial commenced, with reasons

Does your paper address CONSORT subitem 6b? *

Copy and paste relevant sections from the manuscript (include quotes in quotation marks "like this" to indicate direct quotes from your manuscript), or elaborate on this item by providing additional information not in the ms, or briefly explain why the item is not applicable/relevant for your study

Our trial outcomess have not changed.

7a) How sample size was determined

NPT: When applicable, details of whether and how the clustering by care provides or centers was addressed

7a-i) Describe whether and how expected attrition was taken into account when calculating the sample size

Describe whether and how expected attrition was taken into account when calculating the sample size.

	1	2	3	4	5	
subitem not at all important	○	○	○	●	○	essential

Clear selection

Does your paper address subitem 7a-i?

Copy and paste relevant sections from manuscript title (include quotes in quotation marks "like this" to indicate direct quotes from your manuscript), or elaborate on this item by providing additional information not in the ms, or briefly explain why the item is not applicable/relevant for your study

Your answer

7b) When applicable, explanation of any interim analyses and stopping guidelines

Does your paper address CONSORT subitem 7b? *

Copy and paste relevant sections from the manuscript (include quotes in quotation marks "like this" to indicate direct quotes from your manuscript), or elaborate on this item by providing additional information not in the ms, or briefly explain why the item is not applicable/relevant for your study

This study used post hoc analysis, and all analyses were conducted after the end of the study.

8a) Method used to generate the random allocation sequence
NPT: When applicable, how care providers were allocated to each trial group

Does your paper address CONSORT subitem 8a? *

Copy and paste relevant sections from the manuscript (include quotes in quotation marks "like this" to indicate direct quotes from your manuscript), or elaborate on this item by providing additional information not in the ms, or briefly explain why the item is not applicable/relevant for your study

"Randomization was achieved using a computer-generated process. We used Python's random library to generate random numbers for each participant. Specifically, the random.randint(1, 100) function assigned a random integer between 1 and 100 to each patient. Patients with numbers 1-50 were assigned to the treatment group, while those with numbers 51-100 were assigned to the control group."

8b) Type of randomisation; details of any restriction (such as blocking and block size)

Does your paper address CONSORT subitem 8b? *

Copy and paste relevant sections from the manuscript (include quotes in quotation marks "like this" to indicate direct quotes from your manuscript), or elaborate on this item by providing additional information not in the ms, or briefly explain why the item is not applicable/relevant for your study

We don't have any information about Type of randomization; Details of any restriction report.

9) Mechanism used to implement the random allocation sequence (such as sequentially numbered containers), describing any steps taken to conceal the sequence until interventions were assigned

Does your paper address CONSORT subitem 9? *

Copy and paste relevant sections from the manuscript (include quotes in quotation marks "like this" to indicate direct quotes from your manuscript), or elaborate on this item by providing additional information not in the ms, or briefly explain why the item is not applicable/relevant for your study

"Randomization was achieved using a computer-generated process. We used Python's random library to generate random numbers for each participant. Specifically, the random.randint(1, 100) function assigned a random integer between 1 and 100 to each patient. Patients with numbers 1-50 were assigned to the treatment group, while those with numbers 51-100 were assigned to the control group."

10) Who generated the random allocation sequence, who enrolled participants, and who assigned participants to interventions

Does your paper address CONSORT subitem 10? *

Copy and paste relevant sections from the manuscript (include quotes in quotation marks "like this" to indicate direct quotes from your manuscript), or elaborate on this item by providing additional information not in the ms, or briefly explain why the item is not applicable/relevant for your study

This study has one main researcher responsible for related matters, "This study has one main researcher responsible for related matters".

11a) If done, who was blinded after assignment to interventions (for example, participants, care providers, those assessing outcomes) and how

NPT: Whether or not administering co-interventions were blinded to group assignment

11a-i) Specify who was blinded, and who wasn't

Specify who was blinded, and who wasn't. Usually, in web-based trials it is not possible to blind the participants [1, 3] (this should be clearly acknowledged), but it may be possible to blind outcome assessors, those doing data analysis or those administering co-interventions (if any).

	1	2	3	4	5	
subitem not at all important	○	○	◉	○	○	essential

Clear selection

Does your paper address subitem 11a-i? *

Copy and paste relevant sections from the manuscript (include quotes in quotation marks "like this" to indicate direct quotes from your manuscript), or elaborate on this item by providing additional information not in the ms, or briefly explain why the item is not applicable/relevant for your study

"The specific process of the trial included a 2-day screening period (varying by hospital), 1 d for surgery, and a 30-day follow-up period (Figure S1). Before surgery, investigators will take participants into a private meeting room for preoperative communication, which, as required, includes the following: the surgical procedure of circumcision, the purpose and necessity of the surgery, and the potential risks associated with the surgery. At the same time, the investigators will instruct the participants on how to correctly fill out the online questionnaire to ensure validity and reliability. All participants were required to return to the room after the circumcision for 30-minute vital signs monitoring. Meanwhile, investigators would provide different manuals based on the assigned groups. Both manuals provided general guidelines on postoperative recovery and strategies for coping with postoperative complications. The contents of both manuals are summarized as follows:"

11a-ii) Discuss e.g., whether participants knew which intervention was the “intervention of interest” and which one was the “comparator”

Informed consent procedures (4a-ii) can create biases and certain expectations - discuss e.g., whether participants knew which intervention was the “intervention of interest” and which one was the “comparator”.

	1	2	3	4	5	
subitem not at all important	○	○	○	●	○	essential

Clear selection

Does your paper address subitem 11a-ii?

Copy and paste relevant sections from the manuscript (include quotes in quotation marks "like this" to indicate direct quotes from your manuscript), or elaborate on this item by providing additional information not in the ms, or briefly explain why the item is not applicable/relevant for your study

Your answer

11b) If relevant, description of the similarity of interventions

(this item is usually not relevant for ehealth trials as it refers to similarity of a placebo or sham intervention to a active medication/intervention)

Does your paper address CONSORT subitem 11b? *

Copy and paste relevant sections from the manuscript (include quotes in quotation marks "like this" to indicate direct quotes from your manuscript), or elaborate on this item by providing additional information not in the ms, or briefly explain why the item is not applicable/relevant for your study

We described the specific contents of the two measures in parallel, but did not describe their correlation because they do not necessarily need to have a correlation relationship.

12a) Statistical methods used to compare groups for primary and secondary outcomes

NPT: When applicable, details of whether and how the clustering by care providers or centers was addressed

Does your paper address CONSORT subitem 12a? *

Copy and paste relevant sections from the manuscript (include quotes in quotation marks "like this" to indicate direct quotes from your manuscript), or elaborate on this item by providing additional information not in the ms, or briefly explain why the item is not applicable/relevant for your study

"Statistical analyses were conducted using SPSS 26.0. All the analyses were based on the intention-to-treat principle. In the analyses of primary outcomes, repeated measures analysis of variance (MANOVA) was used to evaluate the primary outcomes between groups, with time and group as independent variables. Greenhouse-Geisser and Bonferroni corrections were applied where appropriate. Simple effects analysis was used to determine whether the differences in the primary outcomes between the two groups at each time point were significant. Further discussion of the independent risk factors leading to anxiety was conducted using univariate and multivariate linear regression analyses. In the safety outcome analysis, the chi-square test was used to compare the occurrence of AEs. For secondary outcomes, t/U-tests and chi-squared tests were the primary statistical methods employed."

12a-i) Imputation techniques to deal with attrition / missing values

Imputation techniques to deal with attrition / missing values: Not all participants will use the intervention/comparator as intended and attrition is typically high in ehealth trials. Specify how participants who did not use the application or dropped out from the trial were treated in the statistical analysis (a complete case analysis is strongly discouraged, and simple imputation techniques such as LOCF may also be problematic [4]).

	1	2	3	4	5	
subitem not at all important	○	○	○	◉	○	essential

Clear selection

Does your paper address subitem 12a-i? *

Copy and paste relevant sections from the manuscript (include quotes in quotation marks "like this" to indicate direct quotes from your manuscript), or elaborate on this item by providing additional information not in the ms, or briefly explain why the item is not applicable/relevant for your study

We excluded patients with incomplete data.

12b) Methods for additional analyses, such as subgroup analyses and adjusted analyses

Does your paper address CONSORT subitem 12b? *

Copy and paste relevant sections from the manuscript (include quotes in quotation marks "like this" to indicate direct quotes from your manuscript), or elaborate on this item by providing additional information not in the ms, or briefly explain why the item is not applicable/relevant for your study

"Statistical analyses were conducted using SPSS 26.0. All the analyses were based on the intention-to-treat principle. In the analyses of primary outcomes, repeated measures analysis of variance (MANOVA) was used to evaluate the primary outcomes between groups, with time and group as independent variables. Greenhouse-Geisser and Bonferroni corrections were applied where appropriate. Simple effects analysis was used to determine whether the differences in the primary outcomes between the two groups at each time point were significant. Further discussion of the independent risk factors leading to anxiety was conducted using univariate and multivariate linear regression analyses. In the safety outcome analysis, the chi-square test was used to compare the occurrence of AEs. For secondary outcomes, t/U-tests and chi-squared tests were the primary statistical methods employed."

X26) REB/IRB Approval and Ethical Considerations [recommended as subheading under "Methods"] (not a CONSORT item)

X26-i) Comment on ethics committee approval

	1	2	3	4	5	
subitem not at all important	○	○	○	○	◉	essential

Clear selection

Does your paper address subitem X26-i?

Copy and paste relevant sections from the manuscript (include quotes in quotation marks "like this" to indicate direct quotes from your manuscript), or elaborate on this item by providing additional information not in the ms, or briefly explain why the item is not applicable/relevant for your study

Your answer

x26-ii) Outline informed consent procedures

Outline informed consent procedures e.g., if consent was obtained offline or online (how? Checkbox, etc.?), and what information was provided (see 4a-ii). See [6] for some items to be included in informed consent documents.

	1	2	3	4	5	
subitem not at all important	○	○	○	◉	○	essential

Clear selection

Does your paper address subitem X26-ii?

Copy and paste relevant sections from the manuscript (include quotes in quotation marks "like this" to indicate direct quotes from your manuscript), or elaborate on this item by providing additional information not in the ms, or briefly explain why the item is not applicable/relevant for your study

Your answer

X26-iii) Safety and security procedures

Safety and security procedures, incl. privacy considerations, and any steps taken to reduce the likelihood or detection of harm (e.g., education and training, availability of a hotline)

	1	2	3	4	5	
subitem not at all important	○	○	◉	○	○	essential

Clear selection

Does your paper address subitem X26-iii?

Copy and paste relevant sections from the manuscript (include quotes in quotation marks "like this" to indicate direct quotes from your manuscript), or elaborate on this item by providing additional information not in the ms, or briefly explain why the item is not applicable/relevant for your study

Your answer

RESULTS

13a) For each group, the numbers of participants who were randomly assigned, received intended treatment, and were analysed for the primary outcome

NPT: The number of care providers or centers performing the intervention in each group and the number of patients treated by each care provider in each center

Does your paper address CONSORT subitem 13a? *

Copy and paste relevant sections from the manuscript (include quotes in quotation marks "like this" to indicate direct quotes from your manuscript), or elaborate on this item by providing additional information not in the ms, or briefly explain why the item is not applicable/relevant for your study

"From July 1, 2023, to April 29, 2024, a total of 671 participants qualified for inclusion during their first outpatient visit, with 206 excluded for various reasons (Figure 1). Of the 475 participants randomly assigned to the SRT and NET groups, 189 and 199 participants completed the 30-day follow-up and were included, respectively. Approximately 88% of the participants utilized analgesics, and 73% of the participants were followed up using AAC (Figure 1).“

13b) For each group, losses and exclusions after randomisation, together with reasons

Does your paper address CONSORT subitem 13b? (NOTE: Preferably, this is shown in a CONSORT flow diagram) *

Copy and paste relevant sections from the manuscript (include quotes in quotation marks "like this" to indicate direct quotes from your manuscript), or elaborate on this item by providing additional information not in the ms, or briefly explain why the item is not applicable/relevant for your study

”From July 1, 2023, to April 29, 2024, a total of 671 participants qualified for inclusion during their first outpatient visit, with 206 excluded for various reasons (Figure 1). Of the 475 participants randomly assigned to the SRT and NET groups, 189 and 199 participants completed the 30-day follow-up and were included, respectively. Approximately 88% of the participants utilized analgesics, and 73% of the participants were followed up using AAC (Figure 1).”

13b-i) Attrition diagram

Strongly recommended: An attrition diagram (e.g., proportion of participants still logging in or using the intervention/comparator in each group plotted over time, similar to a survival curve) or other figures or tables demonstrating usage/dose/engagement.

	1	2	3	4	5	
subitem not at all important	○	○	○	◉	○	essential

Clear selection

Does your paper address subitem 13b-i?

Copy and paste relevant sections from the manuscript or cite the figure number if applicable (include quotes in quotation marks "like this" to indicate direct quotes from your manuscript), or elaborate on this item by providing additional information not in the ms, or briefly explain why the item is not applicable/relevant for your study

Your answer

14a) Dates defining the periods of recruitment and follow-up

Does your paper address CONSORT subitem 14a? *

Copy and paste relevant sections from the manuscript (include quotes in quotation marks "like this" to indicate direct quotes from your manuscript), or elaborate on this item by providing additional information not in the ms, or briefly explain why the item is not applicable/relevant for your study

This study established recruitment and follow-up periods and presented them in the form of figures.

14a-i) Indicate if critical “secular events” fell into the study period

Indicate if critical “secular events” fell into the study period, e.g., significant changes in Internet resources available or “changes in computer hardware or Internet delivery resources”

	1	2	3	4	5	
subitem not at all important	(●)	○	○	○	○	essential

Clear selection

Does your paper address subitem 14a-i?

Copy and paste relevant sections from the manuscript (include quotes in quotation marks "like this" to indicate direct quotes from your manuscript), or elaborate on this item by providing additional information not in the ms, or briefly explain why the item is not applicable/relevant for your study

Your answer

14b) Why the trial ended or was stopped (early)

Does your paper address CONSORT subitem 14b? *

Copy and paste relevant sections from the manuscript (include quotes in quotation marks "like this" to indicate direct quotes from your manuscript), or elaborate on this item by providing additional information not in the ms, or briefly explain why the item is not applicable/relevant for your study

This study was completed according to the original plan.

15) A table showing baseline demographic and clinical characteristics for each group

NPT: When applicable, a description of care providers (case volume, qualification, expertise, etc.) and centers (volume) in each group

Does your paper address CONSORT subitem 15? *

Copy and paste relevant sections from the manuscript (include quotes in quotation marks "like this" to indicate direct quotes from your manuscript), or elaborate on this item by providing additional information not in the ms, or briefly explain why the item is not applicable/relevant for your study

Yes.This study presents these results in the form of tables.

15-i) Report demographics associated with digital divide issues

In ehealth trials it is particularly important to report demographics associated with digital divide issues, such as age, education, gender, social-economic status, computer/Internet/ehealth literacy of the participants, if known.

	1	2	3	4	5	
subitem not at all important	○	○	○	○	●	essential

Clear selection

Does your paper address subitem 15-i? *

Copy and paste relevant sections from the manuscript (include quotes in quotation marks "like this" to indicate direct quotes from your manuscript), or elaborate on this item by providing additional information not in the ms, or briefly explain why the item is not applicable/relevant for your study

This study presents these results in the form of tables.

16) For each group, number of participants (denominator) included in each analysis and whether the analysis was by original assigned groups

16-i) Report multiple “denominators” and provide definitions

Report multiple “denominators” and provide definitions: Report N’s (and effect sizes) “across a range of study participation [and use] thresholds” [1], e.g., N exposed, N consented, N used more than x times, N used more than y weeks, N participants “used” the intervention/comparator at specific pre-defined time points of interest (in absolute and relative numbers per group). Always clearly define “use” of the intervention.

	1	2	3	4	5	
subitem not at all important	○	○	◉	○	○	essential

Clear selection

Does your paper address subitem 16-i? *

Copy and paste relevant sections from the manuscript (include quotes in quotation marks "like this" to indicate direct quotes from your manuscript), or elaborate on this item by providing additional information not in the ms, or briefly explain why the item is not applicable/relevant for your study

This study presents these results in the form of tables.

16-ii) Primary analysis should be intent-to-treat

Primary analysis should be intent-to-treat, secondary analyses could include comparing only “users”, with the appropriate caveats that this is no longer a randomized sample (see 18-i).

	1	2	3	4	5	
subitem not at all important	○	○	○	○	◉	essential

Clear selection

Does your paper address subitem 16-ii?

Copy and paste relevant sections from the manuscript (include quotes in quotation marks "like this" to indicate direct quotes from your manuscript), or elaborate on this item by providing additional information not in the ms, or briefly explain why the item is not applicable/relevant for your study

Your answer

17a) For each primary and secondary outcome, results for each group, and the estimated effect size and its precision (such as 95% confidence interval)

Does your paper address CONSORT subitem 17a? *

Copy and paste relevant sections from the manuscript (include quotes in quotation marks "like this" to indicate direct quotes from your manuscript), or elaborate on this item by providing additional information not in the ms, or briefly explain why the item is not applicable/relevant for your study

This study presents these results in the form of tables."Figures 2A-D illustrate primary outcomes measure change over time, specific data is shown in Table S1. These results indicated that time had a significant main effect on the four primary outcomes. GAD-7 scores presented a temporary uptrend firstly and then decline (2.12±0.92 vs 9.95±3.50 vs 5.46±1.32; 2.22±1.14 vs 7.06±2.73 vs 5.34±1.38, F = 920.78, Ptime < 0.01), and NET group had significantly lower GAD-7 scores than those in SRT group at T2 (7.06±2.73 vs. 9.95±3.50, P < 0.05). "

17a-i) Presentation of process outcomes such as metrics of use and intensity of use

In addition to primary/secondary (clinical) outcomes, the presentation of process outcomes such as metrics of use and intensity of use (dose, exposure) and their operational definitions is critical. This does not only refer to metrics of attrition (13-b) (often a binary variable), but also to more continuous exposure metrics such as "average session length". These must be accompanied by a technical description how a metric like a "session" is defined (e.g., timeout after idle time) [1] (report under item 6a).

	1	2	3	4	5	
subitem not at all important	○	○	◉	○	○	essential

Clear selection

Does your paper address subitem 17a-i?

Copy and paste relevant sections from the manuscript (include quotes in quotation marks "like this" to indicate direct quotes from your manuscript), or elaborate on this item by providing additional information not in the ms, or briefly explain why the item is not applicable/relevant for your study

Your answer

17b) For binary outcomes, presentation of both absolute and relative effect sizes is recommended

Does your paper address CONSORT subitem 17b? *

Copy and paste relevant sections from the manuscript (include quotes in quotation marks "like this" to indicate direct quotes from your manuscript), or elaborate on this item by providing additional information not in the ms, or briefly explain why the item is not applicable/relevant for your study

"Result of the multivariable linear regression using these seven factors was presented in Table 3. Grouping (beta = -0.38, 95% CI -3.15 to -2.05, P < 0.01), ORT recall (beta = -0.10, 95% CI -2.15 to -0.33, P < 0.01), swelling reported on the 14th postoperative day (beta = 0.46, 95% CI 2.604 to 3.654, P < 0.01), hematoma (beta = 0.25, 95% CI 1.22 to 2.27, P < 0.01), and NRS scores (beta = 0.10, 95% CI 0.05 to 0.38, P = 0.01) were identified as independent risk factors for GAD-7 scores (T3) (Table 2)."

18) Results of any other analyses performed, including subgroup analyses and adjusted analyses, distinguishing pre-specified from exploratory

Does your paper address CONSORT subitem 18? *

Copy and paste relevant sections from the manuscript (include quotes in quotation marks "like this" to indicate direct quotes from your manuscript), or elaborate on this item by providing additional information not in the ms, or briefly explain why the item is not applicable/relevant for your study

"The secondary outcomes were the use of analgesics and two follow-up methods. Satisfaction scores were measured using a five-point Likert scale, with higher scores (1-5 points) indicating greater satisfaction. The time spent by the staff to reply to messages was documented using the backend statistical system. We assessed the risk of OUD for all participants with Opioid Risk Tool (ORT). Recall of the ORT involved asking during the last online follow-up, "What was your level of OUD risk: [low, medium, high]", and comparing this with the baseline consistency[35]. The safety outcomes were adverse events (AEs), including edema, bleeding, and infection, until 30 days after surgery."

18-i) Subgroup analysis of comparing only users

A subgroup analysis of comparing only users is not uncommon in ehealth trials, but if done, it must be stressed that this is a self-selected sample and no longer an unbiased sample from a randomized trial (see 16-iii).

	1	2	3	4	5	
subitem not at all important	○	○	○	◉	○	essential

Clear selection

Does your paper address subitem 18-i?

Copy and paste relevant sections from the manuscript (include quotes in quotation marks "like this" to indicate direct quotes from your manuscript), or elaborate on this item by providing additional information not in the ms, or briefly explain why the item is not applicable/relevant for your study

Your answer

19) All important harms or unintended effects in each group
(for specific guidance see CONSORT for harms)

Does your paper address CONSORT subitem 19? *

Copy and paste relevant sections from the manuscript (include quotes in quotation marks "like this" to indicate direct quotes from your manuscript), or elaborate on this item by providing additional information not in the ms, or briefly explain why the item is not applicable/relevant for your study

No, the intervention measures in this study do not involve harm or unintended effects.

19-i) Include privacy breaches, technical problems

Include privacy breaches, technical problems. This does not only include physical “harm” to participants, but also incidents such as perceived or real privacy breaches [1], technical problems, and other unexpected/unintended incidents. “Unintended effects” also includes unintended positive effects [2].

	1	2	3	4	5	
subitem not at all important	○	○	◉	○	○	essential

Clear selection

Does your paper address subitem 19-i?

Copy and paste relevant sections from the manuscript (include quotes in quotation marks "like this" to indicate direct quotes from your manuscript), or elaborate on this item by providing additional information not in the ms, or briefly explain why the item is not applicable/relevant for your study

Your answer

19-ii) Include qualitative feedback from participants or observations from staff/researchers

Include qualitative feedback from participants or observations from staff/researchers, if available, on strengths and shortcomings of the application, especially if they point to unintended/unexpected effects or uses. This includes (if available) reasons for why people did or did not use the application as intended by the developers.

	1	2	3	4	5	
subitem not at all important	○	○	○	○	◉	essential

Clear selection

Does your paper address subitem 19-ii?

Copy and paste relevant sections from the manuscript (include quotes in quotation marks "like this" to indicate direct quotes from your manuscript), or elaborate on this item by providing additional information not in the ms, or briefly explain why the item is not applicable/relevant for your study

Your answer

DISCUSSION

22) Interpretation consistent with results, balancing benefits and harms, and considering other relevant evidence

NPT: In addition, take into account the choice of the comparator, lack of or partial blinding, and unequal expertise of care providers or centers in each group

22-i) Restate study questions and summarize the answers suggested by the data, starting with primary outcomes and process outcomes (use)

Restate study questions and summarize the answers suggested by the data, starting with primary outcomes and process outcomes (use).

	1	2	3	4	5	
subitem not at all important	○	○	○	◉	○	essential

Clear selection

Does your paper address subitem 22-i? *

Copy and paste relevant sections from the manuscript (include quotes in quotation marks "like this" to indicate direct quotes from your manuscript), or elaborate on this item by providing additional information not in the ms, or briefly explain why the item is not applicable/relevant for your study

"In this randomized controlled double-blind multicenter trial, we found that patients experienced anxiety mood after VMMC due to pain and adverse events. In contrast to participants who received standardized postoperative communication, those who received narrative communication had lower GAD-7 scores, higher PSQI scores, and improved quality of life within 30-day post-surgery. Furthermore, participants in NET group performed better in the analysis set for analgesic medication use. Moreover, as an exploratory method, AI-assisted follow-up significantly reduced the time healthcare workers spent on follow-up tasks."

22-ii) Highlight unanswered new questions, suggest future research

Highlight unanswered new questions, suggest future research.

	1	2	3	4	5	
subitem not at all important	○	○	○	◉	○	essential

Clear selection

Does your paper address subitem 22-ii?

Copy and paste relevant sections from the manuscript (include quotes in quotation marks "like this" to indicate direct quotes from your manuscript), or elaborate on this item by providing additional information not in the ms, or briefly explain why the item is not applicable/relevant for your study

Your answer

20) Trial limitations, addressing sources of potential bias, imprecision, and, if relevant, multiplicity of analyses

20-i) Typical limitations in ehealth trials

Typical limitations in ehealth trials: Participants in ehealth trials are rarely blinded. Ehealth trials often look at a multiplicity of outcomes, increasing risk for a Type I error. Discuss biases due to non-use of the intervention/usability issues, biases through informed consent procedures, unexpected events.

	1	2	3	4	5	
subitem not at all important	○	○	○	◉	○	essential

Clear selection

Does your paper address subitem 20-i? *

Copy and paste relevant sections from the manuscript (include quotes in quotation marks "like this" to indicate direct quotes from your manuscript), or elaborate on this item by providing additional information not in the ms, or briefly explain why the item is not applicable/relevant for your study

"This study has certain limitations. First, bias may have been present in the trial procedures. Since this study was conducted across multiple institutions, although there have been attempts to standardize processes before the trial began, the procedures for anesthesia, surgery, and others ultimately depended on the policies of each institution. Although the use of internationally recognized questionnaires improved the generalizability and validity of the results, the process of filling them out remained subjective. "

21) Generalisability (external validity, applicability) of the trial findings

NPT: External validity of the trial findings according to the intervention, comparators, patients, and care providers or centers involved in the trial

21-i) Generalizability to other populations

Generalizability to other populations: In particular, discuss generalizability to a general Internet population, outside of a RCT setting, and general patient population, including applicability of the study results for other organizations

	1	2	3	4	5	
subitem not at all important	○	○	◉	○	○	essential

Clear selection

Does your paper address subitem 21-i?

Copy and paste relevant sections from the manuscript (include quotes in quotation marks "like this" to indicate direct quotes from your manuscript), or elaborate on this item by providing additional information not in the ms, or briefly explain why the item is not applicable/relevant for your study

Your answer

21-ii) Discuss if there were elements in the RCT that would be different in a routine application setting

Discuss if there were elements in the RCT that would be different in a routine application setting (e.g., prompts/reminders, more human involvement, training sessions or other co-interventions) and what impact the omission of these elements could have on use, adoption, or outcomes if the intervention is applied outside of a RCT setting.

	1	2	3	4	5	
subitem not at all important	○	○	◉	○	○	essential

Clear selection

Does your paper address subitem 21-ii?

Copy and paste relevant sections from the manuscript (include quotes in quotation marks "like this" to indicate direct quotes from your manuscript), or elaborate on this item by providing additional information not in the ms, or briefly explain why the item is not applicable/relevant for your study

Your answer

OTHER INFORMATION

23) Registration number and name of trial registry

Does your paper address CONSORT subitem 23? *

Copy and paste relevant sections from the manuscript (include quotes in quotation marks "like this" to indicate direct quotes from your manuscript), or elaborate on this item by providing additional information not in the ms, or briefly explain why the item is not applicable/relevant for your study

Yes, registration information has been added to this study.

24) Where the full trial protocol can be accessed, if available

Does your paper address CONSORT subitem 24? *

Cite a Multimedia Appendix, other reference, or copy and paste relevant sections from the manuscript (include quotes in quotation marks "like this" to indicate direct quotes from your manuscript), or elaborate on this item by providing additional information not in the ms, or briefly explain why the item is not applicable/relevant for your study

Due to institutional limitations, the full trial protocol has not yet been made public.

25) Sources of funding and other support (such as supply of drugs), role of funders

Does your paper address CONSORT subitem 25? *

Copy and paste relevant sections from the manuscript (include quotes in quotation marks "like this" to indicate direct quotes from your manuscript), or elaborate on this item by providing additional information not in the ms, or briefly explain why the item is not applicable/relevant for your study

The funding information of this study has been elaborated in detail in the "Funding" section of the manuscript.

X27) Conflicts of Interest (not a CONSORT item)

X27-i) State the relation of the study team towards the system being evaluated

In addition to the usual declaration of interests (financial or otherwise), also state the relation of the study team towards the system being evaluated, i.e., state if the authors/evaluators are distinct from or identical with the developers/sponsors of the intervention.

	1	2	3	4	5	
subitem not at all important	○	○	○	◉	○	essential

Clear selection

Does your paper address subitem X27-i?

Copy and paste relevant sections from the manuscript (include quotes in quotation marks "like this" to indicate direct quotes from your manuscript), or elaborate on this item by providing additional information not in the ms, or briefly explain why the item is not applicable/relevant for your study

Your answer

About the CONSORT EHEALTH checklist

As a result of using this checklist, did you make changes in your manuscript? *

- ○ yes, major changes
- ◉ yes, minor changes
- ○ no

What were the most important changes you made as a result of using this checklist?

Your answer

How much time did you spend on going through the checklist INCLUDING making changes in your manuscript *

I spent a total of 5 hours making these modifications.

As a result of using this checklist, do you think your manuscript has improved? *

- ◉ yes
- ○ no
- ○ Other:

Would you like to become involved in the CONSORT EHEALTH group?

This would involve for example becoming involved in participating in a workshop and writing an "Explanation and Elaboration" document

- ( ) yes
- (x) no
- ( ) Other:

Clear selection

Any other comments or questions on CONSORT EHEALTH

Your answer

STOP - Save this form as PDF before you click submit

To generate a record that you filled in this form, we recommend to generate a PDF of this page (on a Mac, simply select "print" and then select "print as PDF") before you submit it.

When you submit your (revised) paper to JMIR, please upload the PDF as supplementary file.

Don't worry if some text in the textboxes is cut off, as we still have the complete information in our database. Thank you!

Final step: Click submit !

Click submit so we have your answers in our database!

Submit

Clear form

Never submit passwords through Google Forms.

This content is neither created nor endorsed by Google. - Contact form owner - Terms of Service - Privacy Policy

Does this form look suspicious? Report

Google Forms
